# New insights in oocyte dynamics shed light on the complexities associated with fish reproductive strategies

**DOI:** 10.1038/s41598-019-54672-3

**Published:** 2019-12-05

**Authors:** Alba Serrat, Fran Saborido-Rey, Cristina Garcia-Fernandez, Marta Muñoz, Josep Lloret, Anders Thorsen, Olav Sigurd Kjesbu

**Affiliations:** 1 Marine Resources and Biodiversity (GR MAR), University of Girona, 17003 Girona, Spain; 2 Department of Ecology and Marine Resources, Institute of Marine Research (IIM-CSIC), 36208 Vigo, Spain; 3 Institute of Marine Research (IMR), N-5817 Bergen, Norway

**Keywords:** Ecophysiology, Marine biology

## Abstract

Information on temporal variations in stock reproductive potential (SRP) is essential in fisheries management. Despite this relevance, fundamental understanding of egg production variability remains largely unclear due to difficulties in tracking the underlying complex fluctuations in early oocyte recruitment that determines fecundity. We applied advanced oocyte packing density theory to get in-depth, quantitative insights across oocyte stages and seasons, selecting the commercially valuable European hake (*Merluccius merluccius*) as a case study. Our work evidenced sophisticated seasonal oocyte recruitment dynamics and patterns, mostly driven by a low-cost predefinition of fecundity as a function of fish body size, likely influenced also by environmental cues. Fecundity seems to be defined at a much earlier stage of oocyte development than previously thought, implying a quasi-determinate – rather than indeterminate – fecundity type in hake. These results imply a major change in the conceptual approach to reproductive strategies in teleosts. These findings not only question the current binary classification of fecundity as either determinate or indeterminate, but also suggest that current practices regarding potential fecundity estimation in fishes should be complemented with studies on primary oocyte dynamics. Accordingly, the methodology and approach adopted in this study may be profitably applied for unravelling some of the complexities associated with oocyte recruitment and thereby SRP variability.

## Introduction

In modern natural resource management, maintaining a population’s reproductive potential above a certain minimum threshold value (using a proxy, such as the spawning stock biomass in the case of fisheries) is one of the most important practical components of sustainability plans for populations and ecosystems services^1,2^. In fish populations, recruitment results from a myriad of interactions, beginning with factors that determine the level of egg production^3^ which, in turn, can be traced back to fundamental processes influencing oogenesis^4,5^. Related parameters – such as fecundity and length of spawning season – are therefore key in defining stock reproductive potential (SRP)^6^ and, consequently, need to be taken into consideration in population dynamics studies^7^. However, properly quantifying the formation of the smallest oocytes (primary oocytes) and thereby better understanding why fecundity varies is a highly complex issue requiring advanced methodology. An added complication in such studies is that, in order to track the fate of the sex cells, the ovarian samples need to be collected over a sufficiently long time-scale to cover the different parts of the reproductive cycle. Furthermore, the impact of environmental stressors and cues on oogenesis can change markedly throughout the year.

The course of oogenesis goes through three main steps: proliferation of oogonia in the lamellar germinal epithelium followed by development of primary oocytes and then secondary oocytes (which ultimately includes ovulation)^8^. The temporal relation between oocyte recruitment from primary to secondary growth and the spawning season defines the fecundity type, which ranges from clearly determinate to indeterminate^9^. In species with determinate fecundity, oocyte recruitment is completed before the onset of the spawning season. This means that potential annual fecundity can, in principle, be estimated by the standing stock of prespawning, secondary growth oocytes since, in the beginning of this phase of development, those oocytes, which will subsequently be released during the spawning season, are stored to be later matured in lots (cohorts). In contrast, indeterminate species are capable of recruiting oocytes to secondary growth throughout the spawning season. Thus, direct estimation of potential annual fecundity is not possible because the total number of oocytes produced per season is not fixed prior to spawning. Instead, annual fecundity is estimated by multiplying typical batch fecundity (number of eggs spawned in a single spawning event) with the number of batches released^10^. Therefore, the appropriate method for estimating egg production depends on the fecundity type of the species in question. However, despite its importance in the reproductive biology of fisheries, the fecundity type – determinate or indeterminate – remains unknown for many species^11^. In this regard it seems that, rather than simply obtaining “snapshots” of secondary growth oocyte development, dedicated studies of oogenesis are needed^12^. Moreover, the question as to whether the fecundity type is genetically predefined, or modulated by habitat and environmental characteristics as an ecophenotypic response, is mostly unresolved^11,13^, which calls into doubt the rigid labelling of species into one type or the other, which is traditionally the case in most marine laboratories. In fact, several studies have already linked fecundity type to geographic distribution^14^, spawning season and the energy allocation strategy during reproduction^15^, suggesting varying degrees of plasticity.

In response to all of these uncertainties regarding basic and applied aspects of identifying fecundity patterns, theoretical and methodological advances have been made over the last decade or so, leading to far more detailed and accurate fecundity studies and also, in several cases, using less labour-intensive methods^11^. The advanced Oocyte Packing Density (OPD) theory^16^, which builds on the digital auto-diametric method^17^ with facets of stereology and volume-based theory, provides a far better understanding of oocyte recruitment, as even the smallest oocytes in different stages can be reliably quantified independently of fecundity type^16,18–20^. Although the use of OPD theory is certainly gaining momentum, it is rightly pointed out that its formulations require special attention by new experimenters if they are to be executed correctly. Such efforts should, however, be balanced against the fact that existing studies on indeterminate species remain scarce, typically providing a limited view of this highly complex oocyte recruitment process, and focusing, for example, mostly on the spawning season, even though teleost reproductive cycles are well known to show circannual periodicity. So, the underlying seasonal oocyte production has, thus far, rarely been adequately addressed quantitatively^18^.

It seems important, therefore, to gain a better understanding of the process of recruitment of the oocytes to be developed during the spawning season, as well as of the factors underlying changes in egg production, both of which are linked to stock productivity^21^. Amongst the long list of external environmental variables possibly involved, photoperiod has been described as one of the most common cues, triggering gonad development in temperate fish species, while factors such as temperature, food availability and physiological status undoubtedly act as drivers, regulating the rate of oocyte recruitment and development^22^. However, in indeterminate species with a protracted spawning season, it quickly becomes extremely complicated to ascertain and isolate the varied roles of these external cues and drivers due to the asynchronies in oocyte development patterns^23^, and the fact that such lengthy experiments are difficult to run under realistic conditions in the laboratory. All things considered, there is a clear need to improve our understanding of the underlying regulation of oocyte recruitment and fecundity pattern, especially in species with an apparent indeterminate fecundity. This requires not only extensive field sampling covering the full reproductive cycle and access to detailed environmental information, but also advanced laboratory routines to track stage-specific oocyte development in reliable, quantitative terms. In this article, we address the fundamental problems of such research and the associated methodologies, in the course of a complex research effort of this kind on the European hake (*Merluccius merluccius*) from the Galician shelf. This species has a highly complicated reproductive strategy but, in general terms, it is among the most data rich species in the world due to its prominent role in the fishing industry as one of the main commercial target species of the French, Spanish and Portuguese fishing fleets in the North Atlantic. A protracted spawning season has been documented including several spawning peaks or seasons within the year^24^. Generally, the species is considered a batch spawner, with an asynchronous oocyte development and continuous indeterminate fecundity^18^. For the southern stock, a high degree of variability in several reproductive traits has been reported^25^; however, certain fundamental aspects of its reproduction are still barely touched upon^24,26^. Altogether, the specific aims of the research described in the present article were (i) to quantify the seasonal dynamics of the whole range of oocyte stages in European hake, applying OPD theory – with special emphasis on early oocyte recruitment, which is widely recognized as a “black box” in the teleost literature; (ii) to re-examine the traditional classification that classifies hake as a species with an indeterminate fecundity pattern; and finally, (iii) to identify potential links between oocyte recruitment and environmental cues, while bearing in mind that multiple cues might be involved.

## Results

### Oocyte development and packing density

**Figure 1:**
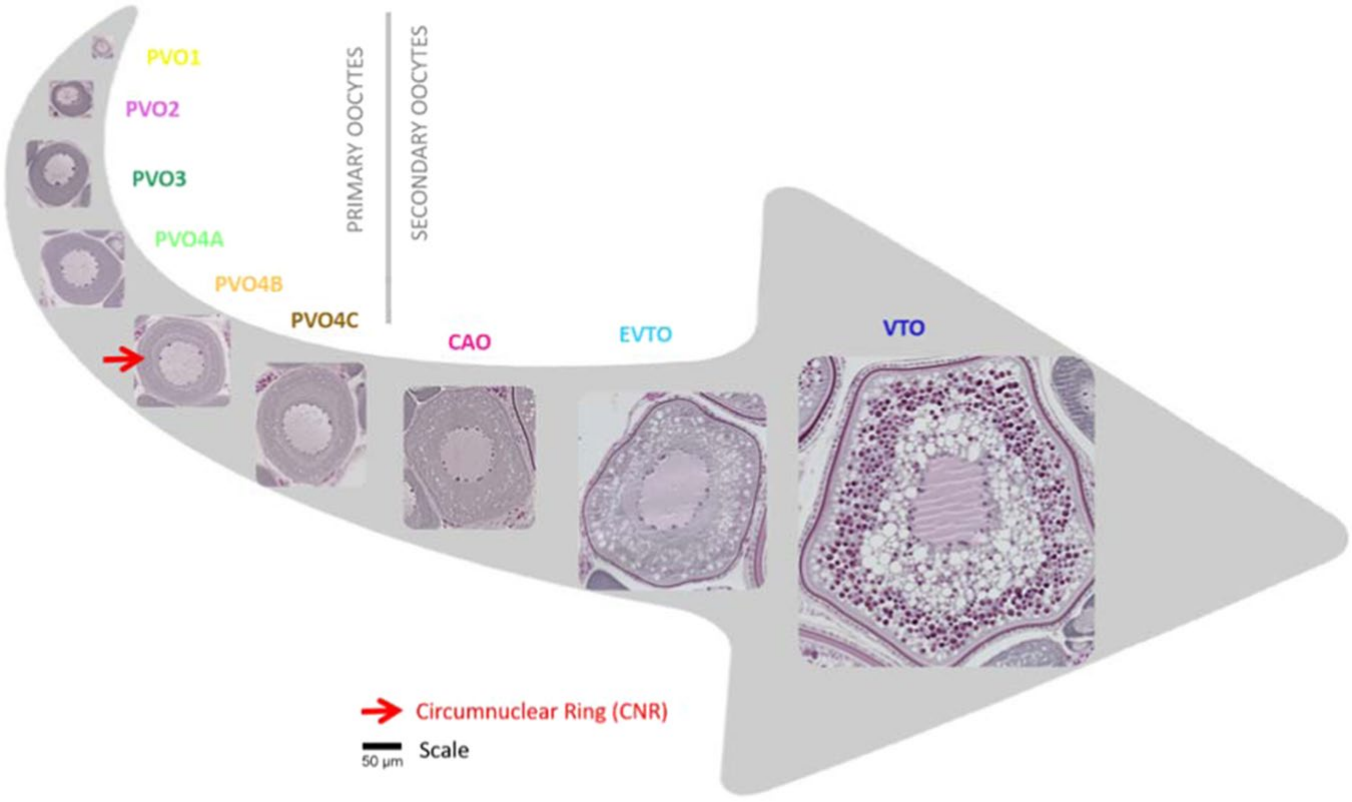
Oocyte stages. Micrographs of various oocyte stages considered for European hake (*Merluccius merluccius*), ranging from previtellogenic oocytes (PVO1-4c) via cortical alveoli oocytes (CAO) to early (EVO) and medium-late vitellogenic oocytes (VTO). PVOs are classified as belonging to primary oocytes, while CAO, EVO and VTO are secondary oocytes. The shape of the illustration mimics the increasing numerical reduction with oocyte stage.

**Figure 2:**
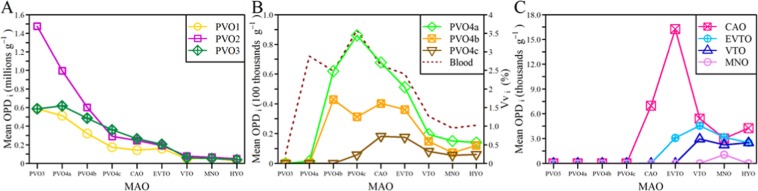
Stage_i_-specific oocyte packing density (OPD_i_). Mean values of OPD_i_ as a function of ovarian development phase (determined by the most advanced oocyte (MAO) present in the ovary) in hake from the Galician shelf from December 2011 to November 2012. The OPD_i_ values are plotted for the following oocyte stages: (**A**) Previtellogenic 1–3: PVO1, PVO2 and PVO3; (**B**) Previtellogenic 4a-c and presence of blood vessels: PVO4a, PVO4b, PVO4c and “blood”; (**C**) Cortical alveoli (CAO), early vitellogenic (EVTO), medium-late vitellogenic oocyte (VTO) and migratory nucleus oocytes. Note that the y-axis scale changes across panels.

Examinations of a series of histological slides (Supplementary, Table S1) confirmed that early oocyte stages of European hake show the same main morphological features as described, for example, for Atlantic cod^27^ and herring^28^. This included the appearance and formation, in the cytoplasm, of the so-called circumnuclear ring (CNR) – which, being rich in organelles and RNA, is homologous with the Balbiani body and, therefore, a central criterion for staging these oocytes (Fig. 1 and Supplementary, Table S2). The oocyte packing density (OPD) is the number of oocytes per gram of ovary. The OPD for each oocyte stage (OPD_i_) manifested markedly different patterns as a function of ovarian growth – or more specifically, the ovarian phase defined by the presence of the most advanced oocyte stage (MAO) (Fig. 2). The smallest previtellogenic oocytes, PVO1, 2 and 3 (Supplementary, Table S2), showed the highest densities (in millions g^−1^) at earlier ovarian phases, but then steadily declined towards spawning (Fig. 2A). In contrast, being larger in size, the PVO4a-c (Supplementary Table S2), showed a right-skewed, dome-shaped density pattern (in hundred thousands g^−1^), starting off with a sharp increase when MAO approached secondary growth (Fig. 2B). Similar patterns (but now counted in thousands g^−1^) were observed for subsequent secondary growth oocytes, i.e. in the stages defined by the presence of cortical alveoli (CAO), vitellogenic (EVO and VTO), then migrating nucleus (MNO) and finally, hydrating oocytes (HYO) (Fig. 2C and Supplementary, Table S2). While PVO1–3 oocytes were omnipresent, all other subsequent oocyte stages (from PVO4a to MNO-HYO) appeared in the ovary associated with the process of “gonad ripening”, each peaking at different ovarian phases, although always detectable until completion of spawning, as is typical for an asynchronous species. These OPD_i_ values fluctuated in successive, overlapping waves, decreasing in presence, with the sharpest numerical reduction happening from oocyte stages PVO3 to PVO4a (Fig. 2A,B) and from PVO4a to PVO4b (Fig. 2B), referring in both cases to ovarian phase PVO4c. The amount of blood capillaries in the ovary (given by volume fraction; Vv_i_) showed a pattern similar to PVO4a and PVO4b oocytes (Fig. 2B). Ovaries in the regenerating phase, i.e. with spawning markers (see below), always contained PVO4b. However, we also found examples of ovaries in the PVO4b phase without any traces of previous spawning, indicating females in puberty; these were observed among specimens of <55 cm in total length (TL).

### Seasonal quantities of various oocyte stages

The *absolute* number of oocytes in different stages in the whole ovary, found by multiplying OPD_i_ with the corresponding ovary weight adjusted for TL, i.e. relative TL-based NO_i_, was recognized as an effective way forward to pinpoint oocyte production, since individual body size differences (and varying growth dynamics - Supplementary, Fig. S1) across months could be accounted for, thus simplifying result interpretations (Supplementary, Text S1 and Fig. S2). The underlying series of extensive analyses, including also uncorrected and predicted NO_i_ estimates (Supplementary, Figs. S3–S9 and Table S3), largely supported the statements given below. However, as these pilot studies were split into either rather broad TL classes (45–65 and 65–85 cm) or showed less predictive power in the consulted models, any associated examples of possible body size effects are not further detailed, although larger individuals were overall more fecund, as is also dealt with below. A selected sub-set of current NO_i_ results were successfully validated (Supplementary, Fig. S10), in line with evaluations of batch fecundity done previously^29^; we specifically aimed at reducing parameter uncertainty (see Methods, and Supplementary, Figs. S11 and S12).

**Figure 3:**
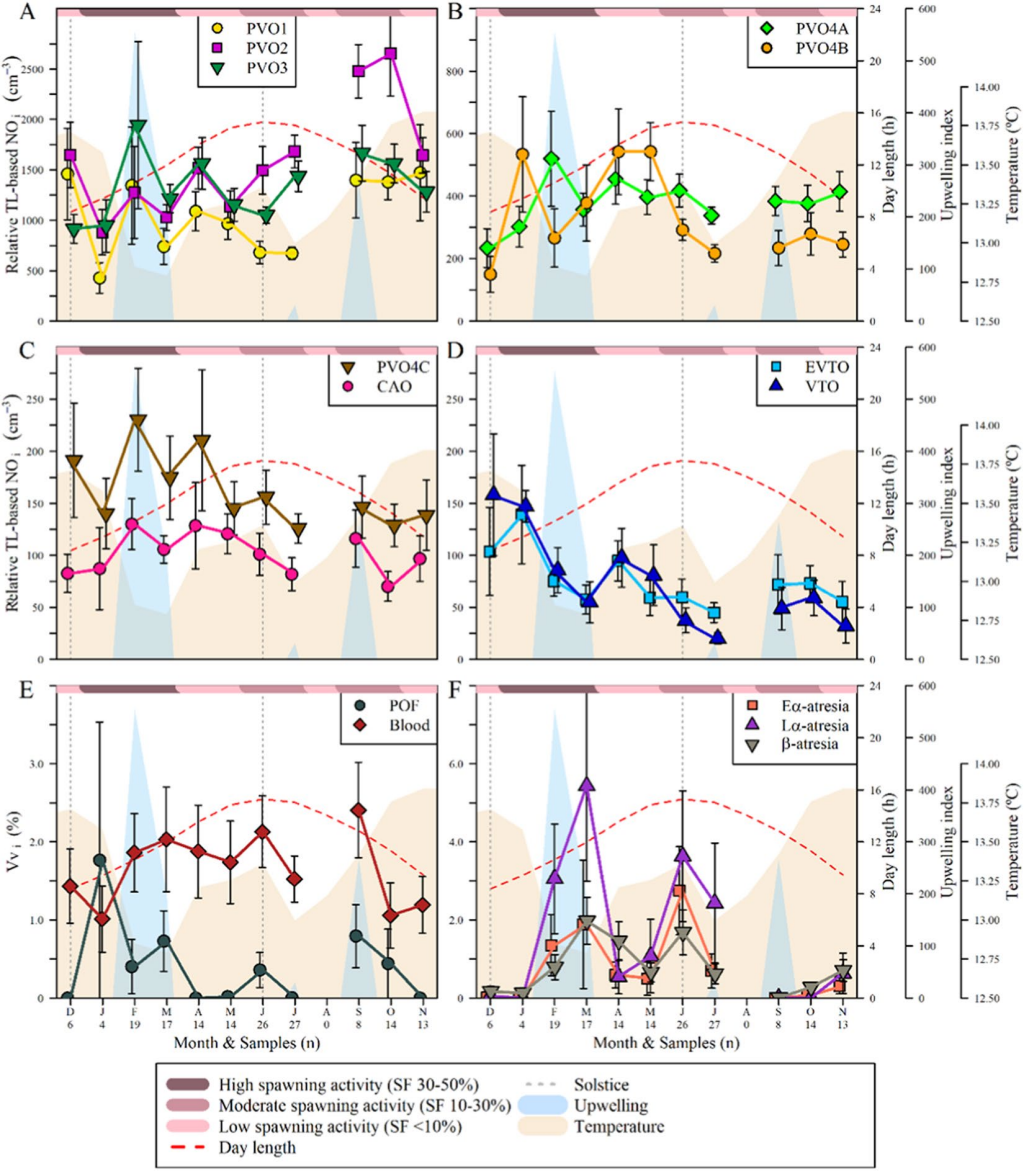
Monthly variation of ovary components. Monthly values (mean ± SE) of relative total length-based stage_i_-specific oocyte number (relative TL-based NO_i_) and volume fraction of different elements in the ovary of hake from the Galician shelf from December 2011 to November 2012. (**A**) Previtellogenic oocyte stages PVO1, PVO2 and PVO3; (**B**) Previtellogenic oocyte stages PVO4a and PVO4b; (**C**) Previtellogenic oocyte stage PVO4c and cortical alveoli oocyte stage CAO; (**D**) Vitellogenic oocyte stages EVTO (early) and VTO (medium and late); (**E**) Amount (volume fraction) of blood vessels (blood) and postovulatory follicles (POF); (**F**) Amount (volume fraction) of early-α (Eα), late-α (Lα) and β atresia. The brown-shaded area corresponds to the mean monthly sea water temperature from 50 to 350 m depth, at 43.5°N 9.5°W, while the blue shaded area shows upwelling events occurring, according to standard definition, when the upwelling index is above zero. The dashed red line shows monthly mean day length, and the dotted grey line marks winter and summer solstice. Spawning activity (at the top of each panel) is given in relation to spawning fraction (SF): 30–50, 10–30 and <10%, in dark, medium and light purple, respectively.

As seen in Fig. 3A, the smallest oocytes (stages PVO1, PVO2 and PVO3), showed similar abundance patterns during the first half of the year, fluctuating around a level of ≈1100 oocytes cm^-3^. However, differences emerged after the summer solstice (and with increasing temperature): PVO2 increased noticeably in number (peaking at ≈2500 oocytes cm^-3^), while PVO1 and PVO3 increased only modestly. Meanwhile, as seen in Fig. 3B, the abundance of PVO4a oocytes showed no clear seasonal trend, whereas PVO4b abundance was roughly twice as high before the summer solstice than it was after it; despite this, overall annual estimates of abundance for PVO4a and PVO4b was fairly similar (≈400 and 325 oocytes cm^−3^, respectively), although this is low compared to the same values for PVO stages 1 to 3 (≈1300 oocytes cm^−3^). The general decline in number continued as the PVOs developed and thereby grew in cell size (Supplementary, Table S2). From PVO4b, to PVO4c, the mean abundance fell by ≈50% (down to ≈160 oocytes cm^−3^) and, as shown in Fig. 3C, by another ≈40% to CAO (down to ≈100 oocytes cm^−3^). The seasonal variation of PVO4c and CAO abundance was roughly similar, with generally higher numbers during winter and spring, more so in the case of PVO4c (cf. ≈180 and 130 oocytes cm^−3^ before and after summer solstice).

With regard to oocytes in early (EVTO) and medium or late vitellogenesis (VTO) (Fig. 3D), typical annual figures of abundance were ≈70 oocytes cm^−3^, i.e. 30% lower than for CAO. For both vitellogenic stages, the lowest numbers were found in July (≈25 oocytes cm^−3^), but there also three concurrent, but diminishing peaks throughout the year. This pattern of oocytic abundance appears to be linked with “pulses” of higher oceanic temperatures and subsequently more-concentrated spawning activity (Supplementary Fig. S13), thereby documenting, in this study, three spawning seasons of European hake within the same year. Also, as shown in Fig. 3E, the volume fraction (Vv_i_) data on postovulatory follicles (POFs) presented three peaks (or possibly four), the first, in January, being the most pronounced, followed by a second smaller peak and a third which was intermediate in size. Finally, in Fig. 3F, which shows the Vv_i_ levels of atresia (sub-grouped into early alpha (Eα), late alpha (Lα) and β stages of atresia), similar patterns can be seen, most noticeably with the Lα stage, although the peaks are delayed compared to the VTO peaks. During the third spawning peak, coinciding with the warmest waters (Supplementary, Fig. S13), atresia was hard to identify histologically (Fig. 3F).

### Oocyte dynamics underlying the act of spawning

**Figure 4:**
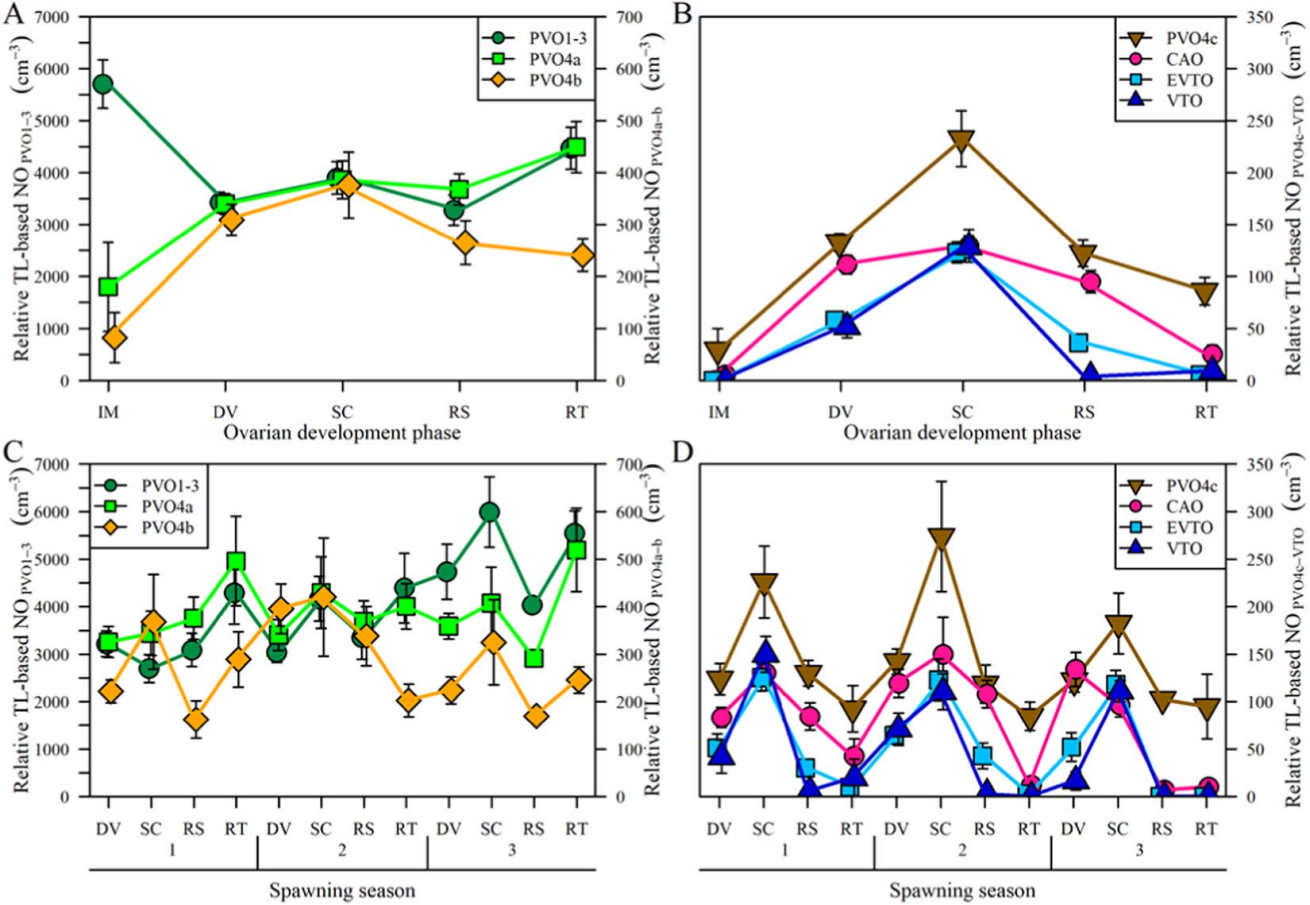
Seasonal variation of oocyte numbers. Relative TL-based NO_i_ (mean ± SE) at different ovarian phases, shown for the whole year (**A,B**) and for spawning seasons 1 (SS1), 2 (SS2) and 3 (SS3) (**C,D**), in hake ovaries collected from the Galician shelf from December 2011 to November 2012. Ovarian phases (Supplementary, Table S1): IM – Immatures; DV – Developing; SC – Spawning Capable; RS: – Regressing; RT: – Regenerating. The AS (actively spawning) phase is included here within the SC category.

Before addressing this topic, it should be noted that data on PVO stages 1, 2 and 3 were pooled, for simplicity, as fluctuations during each of these stages were very similar, with only PVO2 showing a certain degree of deviation (Figs. 2A and 3A). Figure 4 shows the seasonal variation of oocyte numbers at different ovarian phases. Figure 4A shows a high number of oocytes of the PVO1 to 3 stages seen among sexually immature (IM) individuals, but this number falls among mature individuals, and then becomes largely independent of the ovarian phases of developing (DV), spawning capable (SC) and actively spawning (AS) females (AS being pooled with SC). The PVO4a patterns basically match those seen for PVO1-3, except that, rather than a decrease from IM to DV as in the case of PVO1-3 (by ≈2250 oocytes cm^−3^), there was a marked increase (by ≈1750 oocytes cm^−3^). The PVO4b pattern begins to resemble those of the PVO4c to VTO stages, shown in Fig. 4B, which present a spawning-related trend, i.e. increasing from very low values (or absence) among IM individuals to a peak at SC followed by a decrease. This trend might be expected for secondary growth stages (CAO-VTO), but was, in this case, also observed for PVO4b and c, although their number clearly did not fall to such low post-spawning levels as was the case for CAO-VTO. All in all, this type of analysis, split by ovarian phase and ranked by spawning season (Fig. 4C,D), further confirmed the abovementioned evidence of the existence of three clear spawning seasons in European hake, with each spawning season delimited based on spawning fraction, ovarian phase and month of capture (see Methods). The dynamics of the oocyte numbers in stages PVO4b through to VTO in relation to ovarian development were largely similar to those in relation to spawning seasons, with three clear peaks, while PVO1-3 increased along the year, and no obvious trend was seen for PVO4a.

### Resulting fecundity

During the developing and spawning capable ovarian phases, larger females generally had the highest numbers of oocytes (Table 1). For instance, the accumulated number – averaged over spawning seasons – of oocytes, from PVO1-3 stage onwards, was ≈2.1 times higher in females measuring 70–80 cm than for those measuring 50–60 cm. This difference increased to ≈2.6 times when considering only VTO oocytes, suggesting that the resulting batch fecundity was also markedly higher in these bigger specimens (Table 1). However, within each length group, the difference among spawning seasons in the accumulated number of secondary growth oocytes was, on average, rather small, i.e. less than 20%, (including also, in this case, PVO4c, i.e. the most advanced primary oocytes). Hence, the overall egg productivity (or potential fecundity) did not change much across the year, but rather with female size. Nevertheless, the variation in vitellogenic oocytes (EVTO and VTO) was on average > 40% among spawning seasons (especially in medium and large females; Table 1), and this is assumed to reflect clear differences in batch fecundity.

**Table 1:** Analysis of European hake spawning dynamics.

TL range (cm)	Batch fecundity (thousands of oocytes)			Oocyte stage	Potential fecundity			Accumulated number of batches			Spawning season duration (in months)		
	SP1	SP2	SP3		SP1	SP2	SP3	SP1	SP2	SP3	SP1	SP2	SP3
40–50	—	—	142	VTO	—	—	283	—	—	2	—	—	0.3
				+ EVTO	—	—	567	—	—	4	—	—	0.5
				+CA	—	—	850	—	—	6	—	—	0.8
				+PVO4c	—	—	1417	—	—	10	—	—	1.3
				+PVO4b	—	—	1700	—	—	12	—	—	1.6
				+PVO4a	—	—	3401	—	—	24	—	—	3.2
				+PVO1-3	—	—	10202	—	—	72	—	—	9.6
50–60	125	177	128	VTO	249	353	255	2	2	2	0.3	0.3	0.3
				+EVTO	498	706	511	4	4	4	0.5	0.5	0.5
				+CA	995	1059	766	8	6	6	1.1	0.8	0.8
				+PVO4c	1742	1764	1277	14	10	10	1.9	1.3	1.3
				+PVO4b	2737	2823	2042	22	16	16	2.9	2.1	2.1
				+PVO4a	3981	4235	3064	32	24	24	4.3	3.2	3.2
				+PVO1-3	7713	8469	9702	62	48	76	8.3	6.4	10
60–70	306	188	157	VTO	612	375	313	2	2	2	0.3	0.3	0.3
				+EVTO	1225	749	626	4	4	4	0.5	0.5	0.5
				+CA	1837	1498	1253	6	8	8	0.8	1.1	1.1
				+PVO4c	3062	2622	1879	10	14	12	1.3	1.9	1.6
				+PVO4b	4900	4869	3758	16	26	24	2.1	3.5	3.2
				+PVO4a	6737	6742	5323	22	36	34	2.9	4.8	4.5
				+PVO1-3	11637	11985	12525	38	64	80	5.1	8.5	11
70–80	393	290	462	VTO	786	579	924	2	2	2	0.3	0.3	0.3
				+EVTO	1572	1158	1847	4	4	4	0.5	0.5	0.5
				+CA	2357	2317	2771	6	8	6	0.8	1.1	0.8
				+PVO4c	3929	3475	3694	10	12	8	1.3	1.6	1.1
				+PVO4b	7072	6950	5541	18	24	12	2.4	3.2	1.6
				+PVO4a	9430	9845	9236	24	34	20	3.2	4.5	2.7
				+PVO1-3	14930	18532	22166	38	64	48	5.1	8.5	6.4

Females, collected from the Galician shelf from December 2011 to November 2012, were divided into four size categories (TL) and each female assigned to a spawning season. Batch fecundity (BF) was estimated as the average of NO_VTO_ and NO_EVTO_. The number of potential batches at each oocyte stage was estimated as (NO_i_/BF) × 2. The accumulated number of batches, is the sum of batches from oocyte stage VTO backwards to PVO1-3. The potential fecundity was estimated by multiplying batch fecundity and accumulated potential number of batches. The duration of each spawning season was estimated as the product of accumulated batches and spawning frequency, assumed to be typically every four days for all seasons.

To further address fecundity dynamics in European hake, the total number of egg batches a representative individual (within each 10-cm category) could possibly produce over the three spawning seasons in question (SS1 to 3) were estimated theoretically. In the case of 60–70 cm females, this number was 66 (16, 26 and 24 in SS1, SS2 and SS3, respectively), provided all oocytes from PVO4b onwards ended up as eggs (Table 1). However, if we assumed that all oocytes from stages PVO1-3 were dedicated to the current year spawning, the figure would be 182 (38, 64 and 80 for each SS). At the other extreme, only 2 batches per female would be expected in each of the three seasons if the oocytic source in question was limited to the standing pool of VTO (Table 1). Likewise, the associated length of spawning season would range from 0.3 (SS1-3) to 11 months (SS3) (Table 1), with the latter estimate clearly being unrealistic in relation to actual observations (Fig. 3). Similar results were produced for the other length classes that contained sufficient data sets (Table 1).

**Figure 5:**
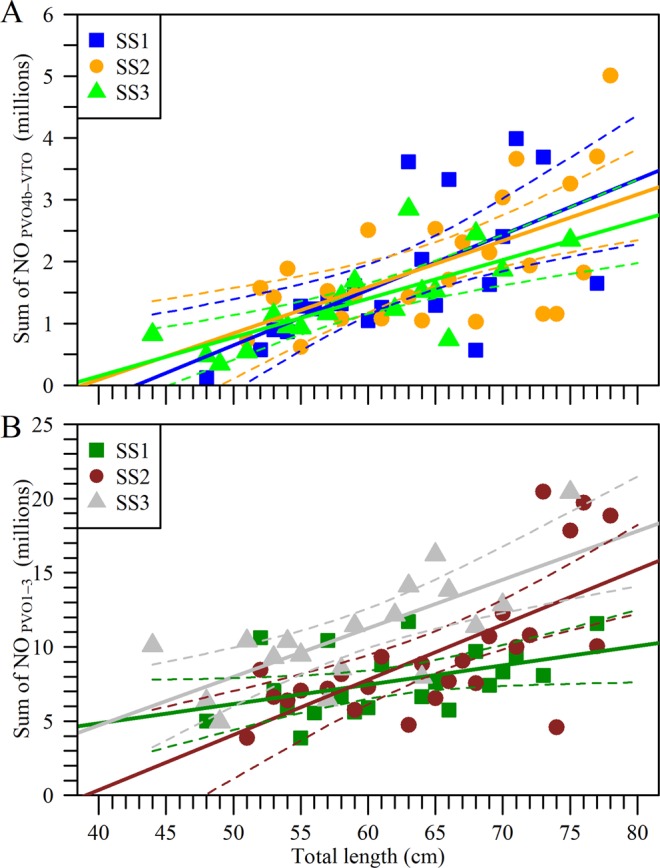
Relationship between number of oocytes and European hake size. (**A**) Sum total of PVO4b, cortical alveoli and vitellogenic (early and medium-late) oocytes (NO_PVO4b-VTO_) regressed against total length; (**B**) Sum of PVO oocytes in stages 1, 2 and 3 (NO_PVO1-3_) regressed against total length. Females were grouped into 1-cm length classes and assigned to one of the three spawning seasons: SS1, SS2 and SS3. Solid line is regression line and dashed lines 95% confidence band.

If we now postulate that European hake has a determinate rather than an indeterminate fecundity pattern, the sum total of CA-VTO numbers should, by default (see standard definitions in the literature), reflect the potential fecundity. A 60–70 cm female, for example, would then produce ≈1.8 million oocytes, spawned in 6 egg batches over 0.8 months in SS1; ≈1.5 million oocytes in 8 egg batches over 1.1 months in SS2; and 1.3 million oocytes in 8 egg batches over 1.1 months in SS3 (Table 1). Yet these estimations are underestimates in relation to published observations, regarding both the expected realized fecundity^24^ and spawning season duration (Fig. 3). However, such calculations become more plausible if we assume that oocytes from the PVO4b stage onwards are to be recruited and spawned during each spawning season (Table 1), and become more unrealistic if our calculations are made from PVO4a onwards. Hence, adopting the total oocyte number from PVO4b to VTO as our proxy for potential fecundity, the relationship with body size (TL) was positive and highly significant (GLM, *P* < 0.001; Fig. 5A), while no differences among spawning seasons was observed (GLM, *P* = 0.76). Interestingly, the total amount of PVO1-3, which are not contributing to annual fecundity, also increased significantly with female size, (GLM, *P* < 0.001) and SS1 showed significant lower values (GLM Post-hoc test, *P* < 0.005) (Fig. 5B).

## Discussion

Oocyte recruitment is influenced by a complex interaction of temporal events and maternal features which, in the case of marine teleosts, have generally received little attention from researchers, and a number of assumptions have therefore been made regarding their reproductive strategies^30^. Here we have shown that oocytes are recruited in hake much earlier than previously thought^22^. This result challenges the classic definitions of determinate and indeterminate tactics^31^ and hence our understanding of fish reproductive strategies. In our opinion, this finding is not exclusive for hake and very likely happens in many, if not most, of the oviparous and pelagophil species. We found that the egg production is mostly dependent on female size, and thereby on female energetics, i.e. their feeding capacity^13^. However, the spawning dynamics differ not only by female size, but also among seasons. The methodology and approach taken in this study appear well suited to throw light on the complexities associated with fish reproductive productivity.

### Method evaluation

State-of-the-art laboratory techniques (the auto-diametric method extended with stereological and volume-based theory) were used to estimate stage-specific oocyte packing density (OPD_i_). This enabled us to make a highly precise exploration of the temporal dynamics of oocyte recruitment, accounting also for the formation of the very smallest primary oocytes, three classes of atresia, post-ovulatory follicles (POFs) as well as the level of other tissue components, including blood capillaries. All in all, the present methods, securely founded on earlier works arising from the introduction of the OPD theory nearly a decade ago^16^, should provide realistic approximations of the seasonal, numerical oocyte production (NO), which is particularly relevant in species with complex reproductive strategies. The results of a previous methodological study on early oocyte ( > 120 µm) recruitment in European hake^18^ cannot be directly compared because a much more detailed staging key for PVOs was used in this study, covering the full yearly cycle instead a part of it. However, in-built validation tests showed a close correspondence between estimated (NO_EVTO_ or NO_VTO_, corrected for shrinkage) and observed values (BF; batch fecundity), the latter having been calculated from whole-mounts^29^. The results indicate that each of these two vitellogenic cohorts will subsequently develop into single batches, thus supporting the use of (NO_EVTO_ + NO_VTO_)/2 as a proxy for batch fecundity.

Oocyte development is a continuous process, which, logically, makes it difficult to always confidently classify stages^4^. Yet, the similarities noted in the dynamics or numerical links between oocyte stages support (as discussed below) the appropriateness of the current oocyte staging scheme, originally applied to cod^27,32^, a determinate spawner with a group-synchronous oocyte development. Here the dynamics of these oocyte stages are evaluated in a teleost displaying an asynchronous oocyte development. Based on our results, sexually immature specimens may be characterized by the presence of PVO3 and PVO4a as the most advanced oocytes (MAO) and small amounts of blood, while ovaries showing oocytes in PVO4b and 4c stages as MAO correspond either to regenerating specimens or specimens at the onset of sexual maturation, i.e. in puberty. This view is supported by previously published results on cod and herring^27,28,32^. Consequently, we argue that the oocyte stage PVO4b, or the final formation of the circumnuclear ring (CNR) represented by PVO4c, could be incorporated in future studies as an early marker of sexual maturation schedules in European hake, and likely in many other teleosts as well. Note, however, that identifying regenerating individuals from those in puberty requires a careful histological check of the presence/absence of actual spawning markers (e.g. POFs)^33^.

### Dynamics of primary oocytes

The major reduction in oocyte numbers between PVO1-3 and PVO4a probably means that there is an actual “reservoir” of the smallest primary oocytes. In other words, this high number of PVO1-3 implies that not all of them will be recruited to be spawned during the current season. Moreover, their temporal, numerical (NO) trend, being opposite to the one seen for vitellogenic oocytes, reinforces the idea that they are not specifically dedicated to the up-coming spawning season(s) but to future ones. We found that this PVO1-3 reservoir is generally built up during the second half of the year, but also that this reservoir is actively utilized in the early transformation from immature to developing ovarian phases. However, the fact that this reservoir is so large complicates any evaluation of direct dynamic links to subsequent oocyte stages. Despite seeing a three-fold decrease in NO values from PVO3 to PVO4a, these two stages followed a similar seasonal pattern independent of spawning activity_._ In contrast, and although the annual mean abundance was similar to that of PVO4a, the PVO4b dynamics were much more – although not completely – related to spawning activity. Furthermore, the PVO4b abundance appeared highest in the first half of the year (after the winter solstice). A second important drop in oocyte numbers was found from PVO4a-b to PVO4c, while from PVO4c to VTO this reduction was more modest and occurred more steadily. As distinct from the postulated long-term reservoir of PVO1-3, these oocytes were obviously being recruited for subsequent spawning, since the abundance of PVO4c onwards, showed three peaks (winter, spring and autumn). Hence, the remaining issue is to elucidate the role of the intermediate or transitional primary stages, PVO4a and PVO4b, which probably have a mixed role as a reservoir and “recruitment spot”. Importantly, their abundance was low in immatures, in contrast to PVO1-3, (note here our above comment that “immatures” might be split between those being true “immatures” and those in early puberty), equally higher in developing and spawning capable females, but, in the case of PVO4b, decreased significantly in regressing and regenerating phases, although far from reaching the zero values observed for vitellogenic oocytes. Hence, our suggestion is that PVO4a should be considered a medium-term reservoir for the current spawning season or for the following one(s); they may or may not be recruited, depending on external and internal factors (see next section). In stark contrast, PVO4b seems closely associated with oocyte recruitment within the current period. Having said that, a precise timing of when and how oocyte recruitment to PVO4b ends was not established in this study, although we did see clear peaks in PVO4b in spawning capable females. In any case, the PVO4b stage should be considered as a pool of cells that can, potentially, be spawned during the up-coming season, in line with the expected realized fecundity and thereby the duration of spawning, as discussed later. The fate of the observed surplus of PVO4a-b at the end of the spawning season remains speculative: these cells might be either broken down through apoptosis or atretic processes^34^, or revitalized as part of next spawning season’s production (see below).

It seems clear, therefore, that the oocytes to be spawned in each spawning season of European hake are “labelled” at some point during the PVO4b stage, but, of course, all these cells will not necessarily make it to the end of the reproductive cycle due to varying incidences of apoptosis or atresia. In this line, the statistical findings of dos Santos Schmidt *et al*.^5^ (although they present no quantitative data on PVOs) clarify that the level of fecundity in herring seems predefined very early on in oogenesis, apparently in response to much earlier feeding opportunities. In our staging system, the PVO4 stages are specifically classified based on the development of the circumnuclear ring (CNR) in cytoplasm, but only the PVO4b stage is characterized by a well-formed and evident CNR. For herring, the appearance of the CNR is likely triggered around the winter solstice, considered to be “the first decision window”, while the spring equinox in this species may operate as “the second decision window”^28^. In fact, photoperiod has been linked to initiation of early oocyte recruitment or development, at least in some high-latitude teleosts where day length, and thereby the strength of this photic signal as an environmental cue, changes markedly throughout the year^32,35^. Here we could not establish any robust relationship between day length and oocyte recruitment, but we did observe a concurrence between the increase in PVO4a and b abundance and the increase in daylight hours after the winter solstice. Consequently, their observed lower numbers during the second half of the year might simply be explained by the subsequent transfer to later developmental stages. However, hake is considered an income breeder^36^, i.e. the acquired energy is immediately used for reproductive investment, meaning there is no need for related, extensive storage^37^. In other words, environmental factors triggering oocyte production (at least vitellogenesis) must necessarily be related with instantaneous energy demands. So, taken together, it seems reasonable to believe that photoperiod may play a potential role in triggering the mobilization of oocytes from “reservoir to recruitment” while the rate at which oocytes develop may be regulated by other environmental factors, e.g. upwelling events attracting foraging fish (the main hake prey)^38^, and the temperature regime experienced^39,40^.

Although a series of gaps exist in our knowledge regarding oogenesis in teleosts, it has been firmly established that oocyte development is stimulated by gonadotropins^4^. However, it is also affirmed that the preceding primary growth (PG) phase is primarily gonadotropin-independent^41,42^, and the production of CAO, the “bridge” between primary growth (PG) and secondary growth (SG), might possibly be related to other hormonal pathways as well^43^. Our results indicate that a spawning-related dynamic is already in place at the PVO4b stage, i.e. well before gonadotropin-dependent regulation. This is a striking finding because it implies that oocyte recruitment (and hence the actual setting of the upper level of potential fecundity) takes place much earlier than traditionally thought. Given the similarity of the oogenesis among fish species^30^, this result has a broad implication in fish reproduction research. Whether this implies that the present focus in the fish fecundity literature on SG rather than PG processes should be reconsidered is a matter of discussion, but given that oocyte recruitment is principally determined during PG, as evident in European hake, the standard definition of determinate and indeterminate fecundity should be, at the very least, revisited. This is because both concepts today relate to oocyte recruitment patterns during SG, or more specifically, during the spawning season as such.

### Egg release

The southern stock of European hake shows a very protracted spawning season, virtually covering the whole year, but with several peaks, as documented here and earlier^24,44^. The observed three peaks or seasons involved different level of spawning activity (relatively high in spring, intermediate in summer and low in autumn), which seems to happen concurrently with upwelling events; egg release during upwelling likely promotes successful reproduction in these types of ecosystems^45^. Also, a protracted spawning season is tightly coupled with an income breeding strategy and indeterminate fecundity^46^; in spawning hake, this is evidenced by intense feeding^47^ and secondary growth oocyte dynamics^48^, respectively. However, our findings challenge today’s thinking: assuming the standing stock of oocytes at PVO4b-VTO stages represents the potential fecundity, the estimations undertaken here give a highly plausible realized fecundity, as would be the case in a determinate fecundity species. Moreover, while we would expect such figures to change between spawning seasons in an indeterminate/income breeder species in accordance with food availability, they actually change with fish size, suggesting strong maternal effects^44^. Note here that production of the smallest oocytes, logically, requires little energy.

Down-regulation of the more advanced oocytes is often seen as a process of matching the available energy^49^. Intense atretic activity was observed both in this and earlier studies on hake^24,50^, but only after the spawning season ceases, with the possible exception of summer time where atresia coincided with the spawning peak. This picture has been linked to an indeterminate strategy^30^, or a “mopping-up” process^51^. Altogether, this supports the impression that down-regulation of fecundity in hake occurs as a consequence of the developmental rate of each cohort (defining the batch fecundity and spawning frequency), which, hypothetically, could be mediated by environmental factors, and which results in the presence of remnants of underdeveloped oocytes to be removed by apoptosis or atresia once the spawning is over.

### Reproductive tactics and strategies

Our results clarify that hake egg productivity (potential fecundity) changes with female size, but not substantially along the year. However, batch fecundity and length of spawning differed significantly by female size and, so far much less documented, also among seasons. These variations are crucial to our understanding of the reproductive strategy, including the realized egg production, of the stock. Temporal and regional variability in reproductive traits related to environmental conditions have been described for several fish species^30,52^. This also applies to the genus *Merluccius*, which displays large phenotypic plasticity in traits such as fecundity and spawning length across stocks inhabiting waters located at different latitudes, including off Galicia^29,53–56^. Our results clearly confirm the existence of three spawning seasons, but spawning frequency could not be directly estimated and was therefore set as constant along the year and with female size (resulting from an averaged individual batch interval of 4 days). Obviously, this constrains our results on spawning duration by season and size class, given by potential/batch fecundity ratios and thereby not specifically attuned to the environmental influences that could certainly affect spawning rhythm.

From a more speculative viewpoint, the findings in this article raise an important, but as yet unresolved, question about the actual duration of spawning or spawning period(s) of a single female within the same year. While this has been assumed to be 2–3 months^24^, it would imply the presence of three spawning components within the same stock. The other option would be that each female takes part in each spawning season, i.e. they present three individual spawning periods, but this would lead to a considerably elevated energetic effort and the longest reproductive season reported for the genus *Merluccius*. Some of our results support this latter idea: i) PVO1-3 increase along the year as if they were to be used later, and ii) the amount of PVO4b-c in regressing and regenerating (RT) females is too high for them to be considered remnants. Although the level of (follicular cell) apoptosis or previtellogenic atresia were not assessed in this study, they are likely to be important processes^34^, at least for PVO1-3 where many of the oocytes “disappeared” to the next stage PVO4a, when contrasting immature and developing ovaries. The PVO4b-c oocytes could serve as surplus for the next spawning period occurring in a matter of weeks, which is supported by the observed dynamics of these PVO stages between RT and SC (spawning capable) females. More specifically, the RT-PVO4b level compares with the SC-PVO4c level, as does the RT-PVO4c level with the SC-VTO level. However, this way of thinking would be based on the idea that hake, living in a regime such as the Galician shelf ecosystem, should be able to spawn over a total of 7–8 months with an annual production of close to 20 million eggs for a 75-cm female. This possibility should not be entirely excluded, e.g. North Sea cod of the same size produced ≈4 million eggs in one single season, but this population of cod then waits a full year for the next season^57^. In any case, another clear option is a much shorter spawning duration for each female hake; it results in a more realistic individual fecundity and reproductive effort, but implies, as stated, the existence of different spawning components, i.e. winter-spring, summer and autumn spawners. Statutory ichthyoplankton surveys since 1999 show that species inhabiting the area in question are either winter or summer spawners, except for the mesopelagic fish, *Maurolicus muelleri*, which has a completely different life strategy, and the present object of study^58^. Also, the presence of three spawning seasons in southern European hake is reasonably well documented through this monitoring program. The existence of stable spawning components suggests some genetic differentiation, since a kind of reproductive isolation must occur. However, this is not a simple issue to address, as shown in genetic analyses conducted on, for example, spring and autumn spawning stocks of herring on both sides of the North Atlantic^59^, where genetic differences between components were certainly identified, but also associated with high gene flow. So, the alternative option of a single component, where each female spawns once a year for a short period, but at different times each year (which would result in three observed seasons) should be considered as biologically unrealistic. Nevertheless, the potential existence of several spawning components within the same stock, or even a single component with several spawning seasons, has a profound implication in fisheries assessment and management. Overfishing of one of the spawning components may occur if not managed properly. On the other hand, the stock-recruitment relationship is a cornerstone in fisheries assessment and estimation of recruitment is dependent on the spawning dynamics. Thus, further research must be performed to support sustainable exploitation.

## Conclusion

We found that, for European hake, oocyte recruitment – with direct consequences for the resulting egg production – occurs much earlier than previously thought, i.e. already during the gonadotropin-independent stage (most likely at PVO4b, but certainly at PVO4c) and that the standing stock of PVO4b-VTO stages reflects reasonably well the potential fecundity of this species. If we adhere to the normal definition of a determinate and an indeterminate pattern, the European hake clearly falls into the latter category, as we found clear evidence of *de novo* oocyte recruitment during spawning (all the way from PVO4b,c to VTO). However, the concept behind this thinking becomes blurred by our findings that the level of fecundity is set much earlier, meaning that hake shows a much more determinate, rather than an indeterminate, fecundity type at the “base line”, which suggests that the definition needs revising. Further to this, early oocyte recruitment patterns clearly differed throughout the year. Our work also points to a complex picture of environmental cues involved. Despite the many issues still unresolved, the fecundity pattern outlined here may well occur in a series of other teleosts. Thus, we question the well-established conceptions of primary and secondary growth oocytes being separate categories rather a continuum, and the concept of determinate versus indeterminate fecundity strategies limited to the spawning season. Hopefully, these results will stimulate new discussions within the scientific community to address teleost reproductive biology, particularly fecundity estimation, in a way that differs from that done today. This may have direct consequences in terms of fisheries advice and management with regard to estimation of SRP and SSB, and thereby potentially help improve sustainability.

## Methods

### Sampling

European hake (*Merluccius merluccius*) females (N = 2961) were collected monthly from commercial gillnet catches from the Galician shelf in the vicinity of the port of Laxe, Spain, from December 2011 to November 2012 (Supplementary Fig. S14). An increased sampling effort was undertaken during seasonal spawning peaks (see main text). Individuals were processed on board or immediately after landing (fishing trips were conducted on a daily basis). Total length (TL, ± 0.1 cm), whole body weight (WW, ± 0.01 g), eviscerated body weight (EW, ± 0.01 g) and gonad weight (GW, ± 0.01 g) were recorded. Macroscopic maturity staging was adopted from standardized terminology^60,61^, and ovaries fixed in 4% buffered formaldehyde for further detailed analyses (N = 162; Supplementary, Table S1). When the main fish data sets were combined with the data from these ovarian tissue subsamples, they provided overviews on the timing and magnitude of egg release (realized egg production), but also gave insights related to the concepts of indeterminacy and determinacy (see below) and, no less importantly, clarified the representativeness of the latter material used in OPD estimation (see below). More specifically, to apply the labour-intensive but highly specific OPD theory method, this subset (34–84 cm TL range) was carefully selected on a seasonal basis, aiming for ten randomly chosen females per ovarian maturity phase (MAT) (see final quality checking below) and each quarter. This scheme, combined with the intensified sampling during spawning seasons, resulted in a number of females per MAT that varied from 9 (immature specimens) to 66 (developing specimens) (Supplementary, Table S1). Once back in the laboratory, formalin-preserved gonad weight (GW_f_, ± 0.01 g) was also registered. Assuming that the ovaries of European hake show an homogeneous oocyte distribution^62^, a cross section of the central part of each ovary was dehydrated, embedded in paraffin and histological sections of 4 µm were cut and stained with haematoxylin and eosin. The MAT was identified microscopically, based on the most advanced oocyte stage (MAO), on whether postovulatory follicles (POF) were present (spawning marker) or not, as well as on the type and amount of atresia, adapted from Hunter and Macewicz^9^ (Supplementary, Table S2).

### Stages of oocyte development

Histological sections were screened in order to classify the stages of oocyte development present (see Fig. 1 for light micrographs and Supplementary, Table S2 for corresponding morphological descriptions), placing special emphasis on detailing the early stages. Hence, primary growth (PG) was divided into four consecutive stages of previtellogenic oocytes (PVO1, PVO2, PVO3, and PVO4) on the basis of cytoplasmic processes, following Shirokova^27^. In line with Shirokova, stage PVO4 was further split into three sub-stages (PVO4a, PVO4b, and PVO4c), specifically based on the location and shape of the circumnuclear ring (CNR), a cytoplasmic structure rich in organelles and RNA (homologous to the Balbiani body). Secondary growth (SG) stages were, following standard procedures, divided into cortical alveoli (CAO), and early (EVTO) and late vitellogenic oocytes (VTO), using zona radiata (chorion) appearance and the degree of accumulation and distribution of yolk granules as criteria, and, ultimately, migratory nucleus (MNO) and hydrated oocytes (HYO)^24^. Atresia was categorized into three types (early alpha: Eα; late alpha: Lα; β: near completion); POFs were also registered^24^. Note: for clarity, we refer to *stages* for oocyte development, and *phases* for ovarian development, the latter being based on MAO, as described above.

### Image analysis

For each ovary, ten micrographs of histological sections (fields) were taken at 10 × magnification, using a digital camera (DFC490 of 8.8 Mpx) mounted on a light microscope (Leica DM500B) with a resolution of 2.33 px/µm. To ensure no overlaps, these photos were taken from every second field starting from the ovarian wall and moving across the section, aided by the motorized Multistep module of the Leica Application Suite. Subsequent image analysis (see below) was carried out using the software ImageJ^63^, and the ObjectJ plugin (https://sils.fnwi.uva.nl/bcb/objectj/). Oocyte size and shape were measured using a slightly modified version of the Elliptical oocytes project (https://sils.fnwi.uva.nl/bcb/objectj/examples/oocytes/), while grid counting was performed using the Weibel Grid Cell project (https://sils.fnwi.uva.nl/bcb/objectj/examples//Weibel/MD/weibel.html).

### Quantification of ovary elements: volume fraction

Several elements of the ovary (including oocyte stages, atresia, POFs, blood vessels (capillaries), missing oocytes and empty space; see Supplementary, Table S2) were quantified, estimating their area fraction, which is proportional to volume fraction (Vv_i_) (according to Delesse’s principle)^64^. The Vv_i_ was computed on grid-overlaid histological sections as the ratio between grid points hitting stage_i_ oocytes (or any of the other mentioned elements) and the total points hitting the sectioned tissue (excluding hits on empty space and outside the ovarian wall). A pilot study was performed in order to establish a compromise between accuracy and time consumption: (i) to avoid underestimation of Vv_i_ of the earliest oocyte stages (PVOs), a first trial was performed on two females to find the most appropriate grid type, using two different set-ups, i.e. Grid *A* (240 points and 77.1 µm probe line length) and Grid *B* (370 points and 65.7 µm probe line length) to estimate Vv_i_, analyzing 10 fields per specimen in each case; (ii) a second trial was performed to define the number of counting fields per sample required to estimate Vv_i_ reliably (given here that we could only use less than 10 fields, see point (i)), aiming at a deviation from normalized mean below ±0.05, studying ovaries from four females (two developing, one spawning capable and one actively spawning) using the selected grid type and ≤10 fields. These methodological issues have already been touched upon by other authors^18,65^, but given that, in this study, we focused on much smaller particles (down to ≈15 µm in oocyte diameter when embedded in paraffin), verification was needed to ensure a good level of precision and accuracy. Regarding trial (i), significant differences in PVO volume fraction appeared between Grid *A* and *B* (*P* < 0.01). Thus, we opted for the most conservative option, i.e. the denser grid (Grid *B*). Regarding the number of fields in trial (ii), the estimated deviation from the normalized mean for every oocyte stage in all specimens analysed stabilized at ±0.05 when seven fields were counted (Supplementary, Fig. S11), indicating that grid counting could be limited to this number of fields.

### Oocyte size and shape

For each specimen (j), short (S) and long (L) axes were measured on 10 oocytes of every oocyte stage (i) in histological slides. Individual oocyte shape factor (k_ij_) was calculated from the ratio between oocyte L and S axis, i.e. k_ij_ = L_ij_/S_ij_, and mean stage_ij_-specific oocyte shape factor thereafter calculated for each female. Only oocytes sectioned through the nucleus were considered for measurements. The above-given number of stage_i_ oocytes to be measured in order to estimate mean stage_i_-specific oocyte shape factor with a deviation from the normalized mean below ±0.05 was defined in a trial test on two females, where up to 15 stage_i_ oocytes were measured (Supplementary, Fig. S12). Individual arithmetic oocyte diameter was calculated as OD_ij_ = (L_ij_ + S_ij_)/2. Stage_i_-specific mean volume-based oocyte diameter was then estimated as OD_vi_ = [Σ^ni^_j=1_(OD_ij)_^3^/n_i_)]^(1/3)^^16^. Due to oocyte shrinkage during histological processing, a correction factor was applied to turn oocyte diameters into their initial, stabilized formaldehyde (formalin)-fixed dimensions, as measured under laboratory conditions in whole mounts (cf. image analysis). We first tested the correction factor developed for resin-embedded ovarian tissue of European hake^66^. However, oocyte shrinkage varies with the embedding medium, being higher in paraffin than resin^65^. Since paraffin was used in this study, a second correction factor from a similar study on *Thunnus alalunga* using paraffin instead, was also taken into account^65^. Hence, we applied both correction factors in order to choose the one that gave the closest fit to the whole-mount recordings. Due to the high degree of shrinkage of hydrated oocytes (HYO) during histological processing and their resulting highly irregular shape, it was considered unfeasible to get reliable measurements of their oocyte size, thus OD_i_ and OPD_i_ were not estimated for this type of oocytes.

### Oocyte packing density

The number of oocytes per gram of ovary, known as the oocyte packing density (OPD)^16^, was estimated for every stage (_i_) of oocytes present in the ovary by applying the refined formula of Korta *et al*.^18^:

1 $$OPDij=\,{\log }[Vvij\times (\frac{1}{\rho o})\times \{{(1+kij)}^{3}/(8\times kij)\}]+12.28-3\times \,{\log }(cODvij)$$

where the variables are defined as follows:

*OPD*_*ij*_: Stage_i_-specific oocyte packing density by female (j)

*Vv*_*ij*_: Volume fraction of stage_i_ oocytes by female (j)

*ρ*_*o*_: Specific gravity of the ovary

*k*_*ij*_: Mean shape factor of stage_i_ oocytes by female (j)

*cODvi*_*i*_: Mean stage_i_ volume-based oocyte diameter by female (j), corrected for shrinkage, cf.^65^.

The specific gravity of the ovary was obtained from Kurita and Kjesbu^16^, i.e. being set at 1.061 and 1.072 for ovaries showing PVO/MNO and CA/EVTO/VTO as the most advanced oocyte stage, respectively.

### Stage_i_ oocyte number estimation

The number of oocytes in stage (_i_) in each ovary (_j_) was calculated from OPD_ij_ and the formalin-fixed gonad weight (GW_fj_) as:

2 $$NOij=OPDij\times GWfj$$

- *Shrinkage correction*. To further clarify which shrinkage correction factor was the best one, we compared recent whole-mount estimates of batch fecundity (BF) for the same stock^29^ with our three estimations of the number of medium-late vitellogenic oocytes (VTO), the first being uncorrected for shrinkage (NOi) and the second and third being corrected for shrinkage using the two correction factors described above (rNOi and cNOi, referring to the resin- and paraffin-embedded correction factor, respectively). The number of VTOs was assumed to reflect BF, as has been previously reported^24^. We found that mean cNO_VTO_ values were closest to BF values at any total length of the female (Supplementary, Fig. S10). Given this result, we adopted the paraffin-embedded correction factor developed for *T. alalunga*^65^ to be used within the current protocol to estimate the total number of oocytes in each stage (hereon labelled as NOi).
- *Approaches for estimating NOi*. As formalin-fixed ovary weight (GWf) (Eq. 2) both varied with ovarian phase (MAT) and female size (TL), this required further consideration when aiming to present the temporal dynamics of NOi in a standardized way. We opted for three approaches: In the first approach, NOi was used without any underlying data transformations, but the analyses were divided into two different female length classes; LC 1: 45–65 cm (i.e. up to 64.9 cm) (n = 101), and LC 2: 65–85 cm (n = 54). In the second approach, we attempted to standardize NOi by body weight directly (relative NOi), preferably, the eviscerated weight (EW) to reduce the confounding effect of varying gonad size. However, in some females, EW data was missing, while TL was always measured, and hence TL was preferred. For those individuals where both metrics were available, EW and TL showed an isometric scaling (Supplementary Fig. S1A), i.e. according to the power function EW = a × TLb (P < 0.001), where b was equal to 3.01 with a standard error of 0.07. In other words, TL^3^ could be used as proxy for EW. Consequently, NOi corrected for size (TL-based NOi) was estimated as NOi/TL^3^. To further validate this second approach, and concentrating on those specimens with EW values, the relative number of oocytes was also calculated as EW-based NOi = NOi/EW. The relationship between TL-based and EW-based NOi was extremely tight (r^2^ = 0.96, F(1,155) = 2555, P < 0.001) (Supplementary Fig. S1B). Finally, the third approach involved modelling the variation in GWf (response variable) statistically, with MAT (categorical factor), TL (continuous variable) and their first order interaction as covariates, i.e. GW = a × MAT + b × TL + c × MAT × TL). This output showed an adjusted r^2^ of 0.62 (F(28.44,9) = 28.44, P < 0.001). Tests on the GW vs. TL relationships demonstrated unequal slopes among MAT (Supplementary, Fig. S15) (F(4,142) = 3.080, P = 0.018, Supplementary Table S3). This set of information was thereafter used to give predicted GWf (pGWf) for each female. Then, the predicted stagei oocyte number (pNOi) was estimated using pGWf in Eq. (2). Finally, the level of independence of female size in each of the three approaches was tested and one of the approaches selected for subsequent analyses in the main study.
- *Temporal trends*. The temporal dynamics of oocyte production was analysed in three ways. First, an overall temporal analysis was conducted by estimating NOi values by month and oocyte stage, including other ovarian elements as well (POF, blood capillaries and atresia). These elements are known to be indicators of ovarian development. The noted variations were compared with trends in environmental variables (daylight duration, temperature and upwelling index), as well as with spawning fraction (SF) (see below). Second, a seasonal analysis was performed by estimating NOi values by ovarian phase (MAT) and oocyte stage within the respective spawning seasons identified. Information on duration and magnitude of spawning peaks was based on monthly SF, estimated as the proportion of the number of actively spawning females and the total number of sexually mature females by month, i.e. an average SF at the population level^44^. Note here that SF was estimated from the complete database of 2961 female specimens. This analysis resulted in the definition of three spawning seasons with different levels of spawning activity (see Supplementary, Fig. S13 for a summarized plot). The same seasons were reported in earlier studies^24,^^25^, and roughly correspond to winter-spring, summer and autumn. To analyse accurately the oocyte dynamic within each spawning season, every female was first assigned to a particular season based on the month of capture and ovarian phase, but considering also the volume fraction of the different oocyte stages, types of atresia, POF and amount of blood vessels. Thus, for example, a female in the regenerating phase captured at the beginning of a spawning season was assumed to have spawned earlier and consequently assigned to the preceding season. In contrast, a female with no signs of spawning activity, e.g. the ovary being in early development, and captured at the end of a spawning season was assumed to spawn later and thus assigned to the next season. Third, this analysis dealt with oocyte recruitment as such, i.e. the manner in which oocytes are recruited from early development stages to spawning, which defines the type of fecundity (indeterminate or determinate), and the spawning dynamics (number of batches, spawning frequency and batch fecundity). To examine the influence of body size on oocyte recruitment dynamics, the analysis was performed using the absolute numbers of NOi (corrected for shrinkage, but non-transformed for female size) while considering four TL ranges (40–50, 50–60, 60–70 and 70–80 cm). For each spawning season, the average NOi by oocyte stage and body size range was computed, pooling females in DV, SC and AS reproductive phases (Table 1). According to the results (see main text), NO_EVTO_ and NO_VTO_ (i.e. oocytes in various parts of vitellogenesis) showed comparable values in a given ovary, irrespective of the spawning season and female size. As we assumed NO_VTO_ as a proxy for batch fecundity, this implied that the joint category of vitellogenic oocytes corresponded, in principle, to two, single batches and a mean batch fecundity was therefore estimated for each spawning season and female size range; BF = (NO_EVTO_ + NO_VTO_)/2. We used this value to estimate the number of batches potentially existing in each oocyte stage and, from this, the average accumulated number of batches produced by a female in each season and size range. However, given that our sampling covered the whole spawning season, and given the strong spawning asynchrony among females (i.e. in a given month, females at every ovarian phase from DV to RG were found; see main text), we assumed that the typical female analysed was in the middle of its spawning period (i.e. while some have recently started to spawn, others were in the middle, and some were almost at the end of the spawning activity). In consequence, each female had already released half of the batches.

### Other sources of information: upwelling, sea water temperature, day and spawning length

Monthly upwelling indexes were provided by the Spanish Institute of Oceanography (www.indicedeafloramiento.ieo.es) using data on sea surface level pressure from the weather research and forecasting (WRF) operational model of Meteogalicia (http://www.meteogalicia.gal) for the region, between December 2011 and November 2012. Day length (number of daylight hours) at 43° 30′N 9°30′W was obtained from the Astronomical Applications Department of the U.S. Naval Observatory Data Service (http://aa.usno.navy.mil/index.php). The sea water temperature at the same geographical position at depth 50–350 m (monthly mean) was acquired from IBIRYS Regional High Resolution Reanalysis^67^ located at Copernicus Marine Environment Monitoring Services (http://www.copernicus.eu/) (product identifier: IBI_ REANALYSIS_PHYS_005_002). The above three selected abiotic factors were specifically used to explore impacts of environmental cues/drivers on European hake oocyte recruitment.

### Statistical analyses

All statistical analyses were performed using R version 3.2.3 (https://www.r-project.org/) with *P* < 0.05 considered as a significant result. Differences in volume fraction (Vvi) between grid types were analyzed using paired t-test. The relationship between TL-based and EW-based NOi, was tested by a linear regression. ANCOVA was applied to contrast slope and intercept values for total length (TL) regressed on formalin-fixed gonad weight (GW_f_) split by ovarian phase (MAT). Relationships between potential fecundity and TL among spawning seasons were assessed by General Linear Models. All tests consulted were executed according to standard routines, as indicated in the various R scripts.

### Ethics statement

No specific permissions for sampling were required for this study as all the individuals sampled were obtained from commercial fishing following the local laws. Fish were purchased once the fishers processed them and analysed on board or right after landing. No protected species were sampled. No authorization or ethics board approval was required to conduct the study.

## Supplementary information

Supplementary information

## Data Availability

The datasets generated and/or analysed during the current study are available in the SEAONE repository, https://www.seanoe.org/data/00427/53820/.
