# Supplementary material for: New insights in oocyte dynamics shed light on the complexities associated with fish reproductive strategies
Source: Sci Rep. 2019 Dec 5;9:18411. doi: 10.1038/s41598-019-54672-3 (PMC6895218; doi:10.1038/s41598-019-54672-3)
Supplement: Supplementary file 1 — Supplementary information [file 41598_2019_54672_MOESM1_ESM.pdf]

Supplementary information

Title: New insights in oocyte dynamics shed light on the complexities associated with fish reproductive strategies

Alba Serrat, Fran Saborido-Rey, Cristina Garcia-Fernandez, Marta Muñoz, Josep Lloret, Anders Thorsen, and Olav Sigurd Kjesbu

This file includes:

- Supplementary figures: Fig S1-S15
- Supplementary tables: Table S1-S3
- Supplementary text to support the results and the discussion

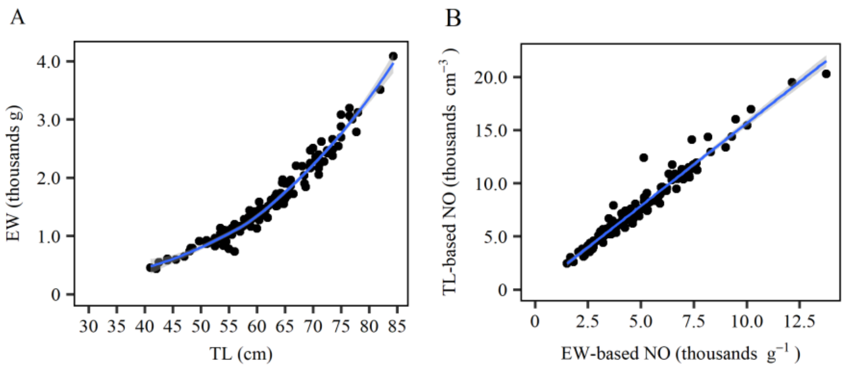

Fig. S1. Relationships between total length (TL) and eviscerated body weight (EW) (A) and TL-based and EW-based oocyte number (NO) (B) of southern European hake. In (B) EW-based NO equals NO/EW, while TL-based NO equals NO/TL3. The solid line is either the fitted power (A), i.e. EW = 0.004 × TL3.103, or the linear regression line (B), where the shaded area in both cases reflects the 95% confidence band.

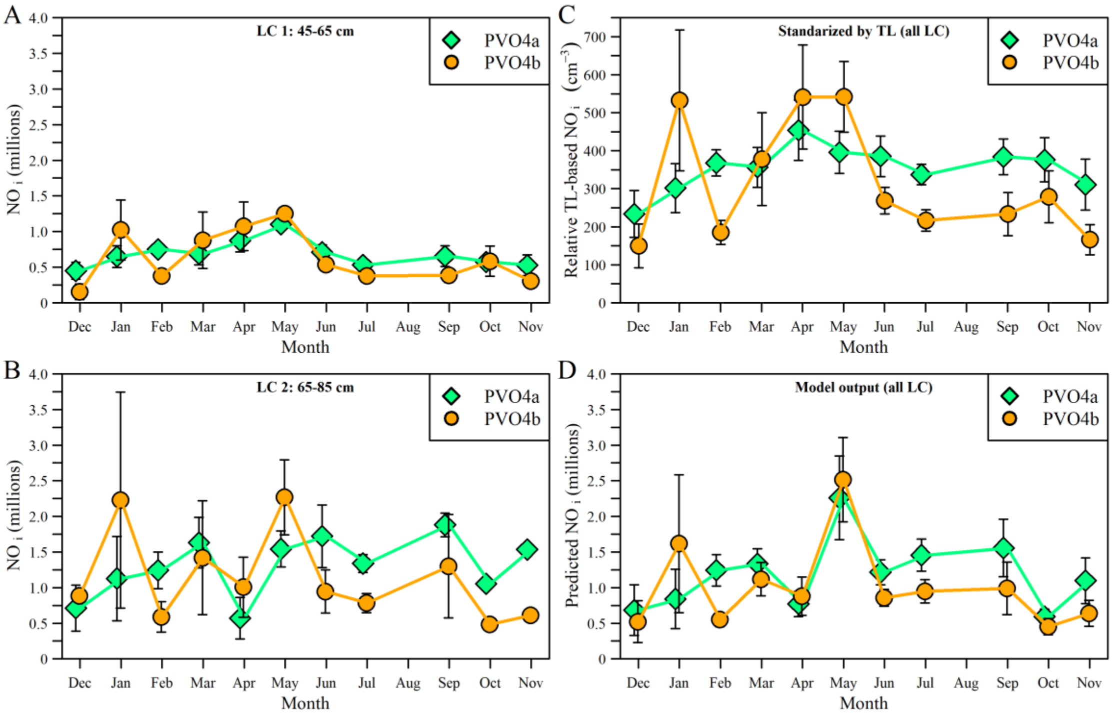

Fig. S2. Approaches to estimate total oocyte number. Estimations of mean number (± SE) of PVO4a and PVO4b oocytes in the ovary of European hake (*Merluccius merluccius*) collected monthly from December 2011 to November 2012 at the Galician shelf (S10 Fig). The number of stage$_i$ oocytes ($NO_i = OPD_i \times GW_f$,) was estimated by three different approaches: (*A*) and (*B*) non-transformed stage$_i$ oocyte number (NO_i), split into two length classes LC1: 45-65 cm and LC2: 65-85 cm; (*C*) relative total length-based number of stage$_i$ oocytes (relative TL-based NO_i); and (*D*) predicted stage$_i$ oocyte number (predicted NO_i), using model output to adjust GW_f for TL and ovarian phase. Missing SE reflects too little data to show these bars. Additional abbreviations: OPD_i: stage$_i$ oocyte packing density; GW_f: formalin-fixed gonad weight.

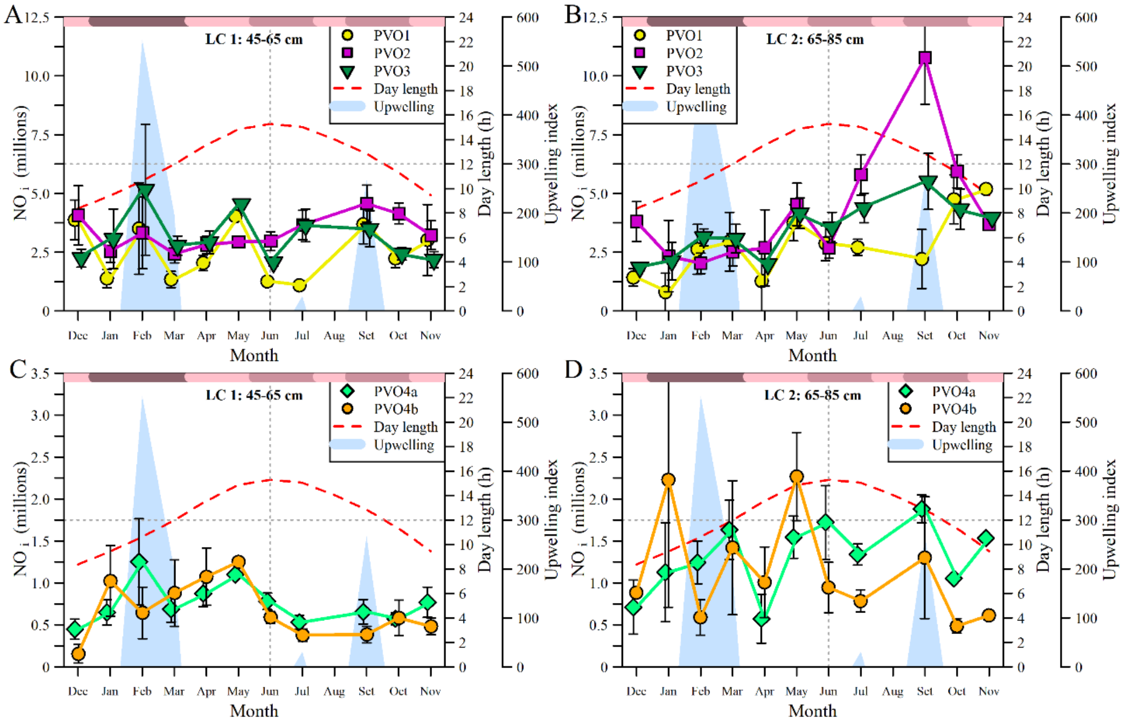

Fig. S3. Previtellogenic oocyte fluctuations, approach 1. Non-transformed $stage_i$ oocyte numbers (NO_i) of (*A* and *B*) previtellogenic 1, 2 and 3 (PVO1, PVO2, PVO3) and (*C* and *D*) previtellogenic 4a and 4b (PVO4a, PVO4b) oocyte stages, split into two total length classes (LC): LC 1, from 45 to 65 cm (*A* and *C*) and LC 2 from 65 to 85 cm (*B* and *D*). Estimates (mean ± SE) refer to data from European hake ovaries collected monthly at the Galician shelf from December 2011 to November 2012. Blue-shaded area shows upwelling events (upwelling index > 0) and dashed line day length. The upper box of the plot summarizes presently noticed spawning activity based on spawning fraction (SF) estimations: dark, medium and light purple corresponds to 30-50, 10-30 and <10% SF, respectively.

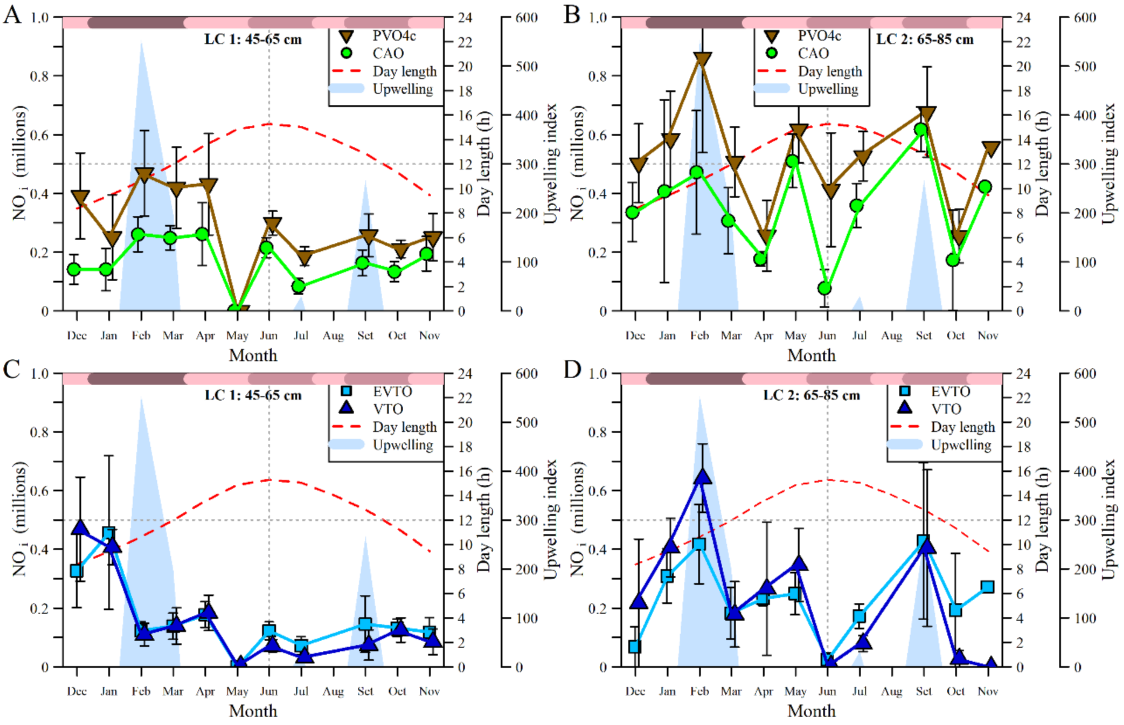

Fig. S4. **Previtellogenic (PVO4c) and secondary growth oocyte fluctuations, approach 1.** Non-transformed stage$_i$ oocyte numbers (NO_i) of (*A* and *B*) previtellogenic 4c (PVO4c) and cortical alveoli oocytes (CAO) and (*C* and *D*) early vitellogenic (EVTO) and medium-late vitellogenic (VTO) oocyte stages, split into two total length classes (LC): LC 1, from 45 to 65 cm (*A* and *C*) and LC 2 from 65 to 85 cm (*B* and *D*). Estimates (mean ± SE) refer to data from European hake ovaries collected monthly at the Galician shelf from December 2011 to November 2012. Blue-shaded area shows upwelling events (upwelling index > 0) and dashed line day length. The upper box of the plot summarizes presently noticed spawning activity based on spawning fraction (SF) estimations: dark, medium and light purple corresponds to 30-50, 10-30 and <10% SF, respectively.

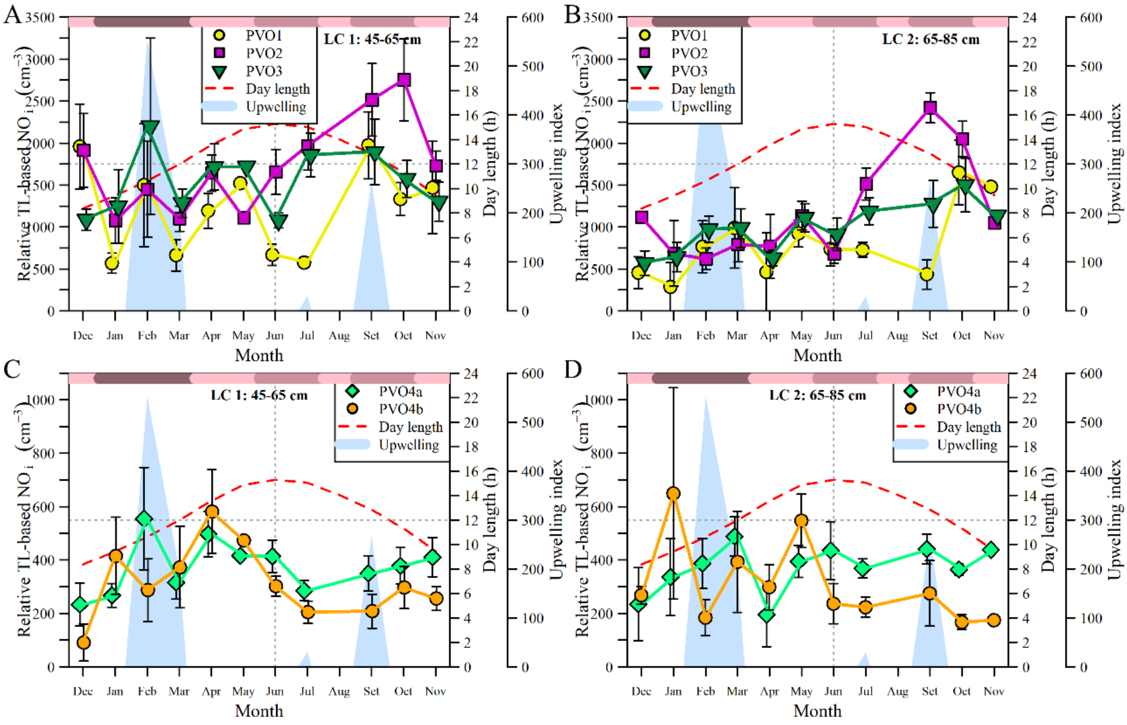

Fig. S5. **Previtellogenic oocyte fluctuations, approach 2.** Relative total length-based stage$_i$ oocyte number (Relative TL-based NO$_i$) for (*A* and *B*) previtellogenic 1, 2 and 3 (PVO1, PVO2, PVO3) and (*C* and *D*) previtellogenic 4a and 4b (PVO4a and PVO4b) oocyte stages, split into two total length classes (LC): LC 1, from 45 to 65 cm (*A* and *C*) and LC 2 from 65 to 85 cm (*B* and *D*). Estimates (mean ± SE) refer to data from European hake ovaries collected monthly at the Galician shelf from December 2011 to November 2012. Blue-shaded area shows upwelling events (upwelling index > 0) and dashed line day length. The upper box of the plot summarizes presently noticed spawning activity based on spawning fraction (SF) estimations: dark, medium and light purple corresponds to 30-50, 10-30 and <10% SF, respectively.

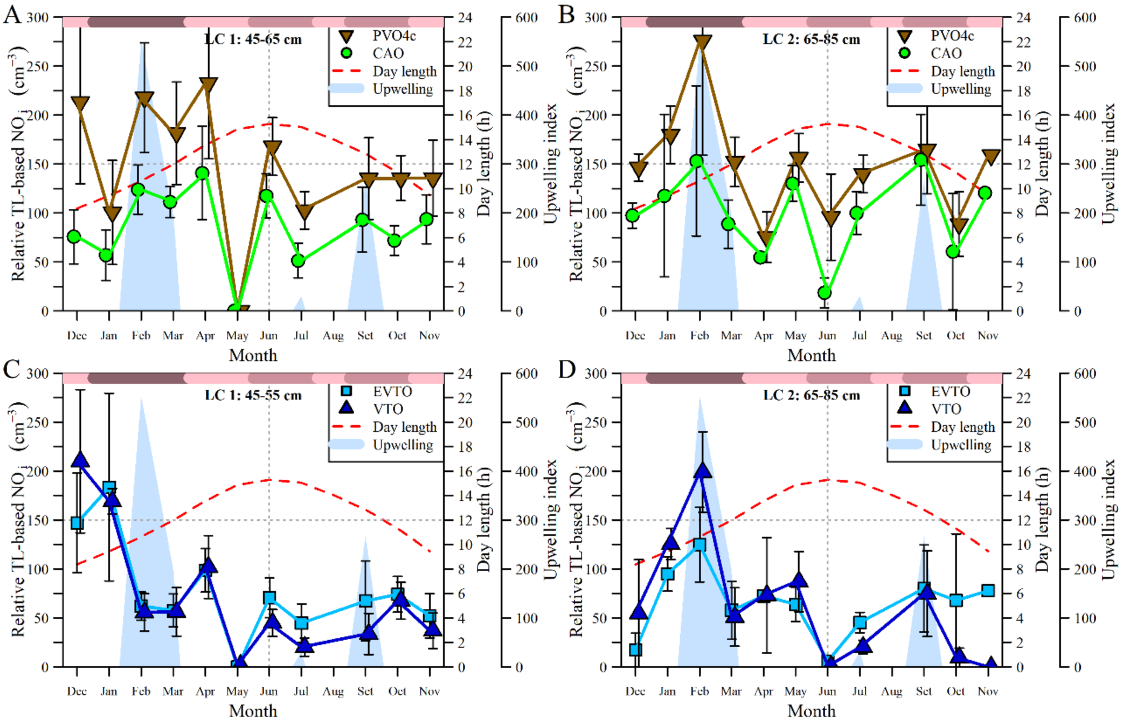

Fig. S6. Previtellogenic (PVO4c) and secondary growth oocyte fluctuations, approach 2. Relative total length-based stage$_i$ oocyte number (Relative TL-based NO$_i$) for (*A* and *B*) previtellogenic 4c and cortical alveoli (PVO4c and CAO) and (*C* and *D*) early vitellogenic (EVTO) and medium-late vitellogenic (VTO) oocyte stages, split into two total length classes (LC): LC 1, from 45 to 65 cm (*A* and *C*) and LC 2 from 65 to 85 cm (*B* and *D*). Estimates (mean ± SE) refer to data from European hake ovaries collected monthly at the Galician shelf from December 2011 to November 2012. Blue-shaded area shows upwelling events (upwelling index > 0) and dashed line day length. The upper box of the plot summarizes presently noticed spawning activity based on spawning fraction (SF) estimations: dark, medium and light purple corresponds to 30-50, 10-30 and <10% SF, respectively.

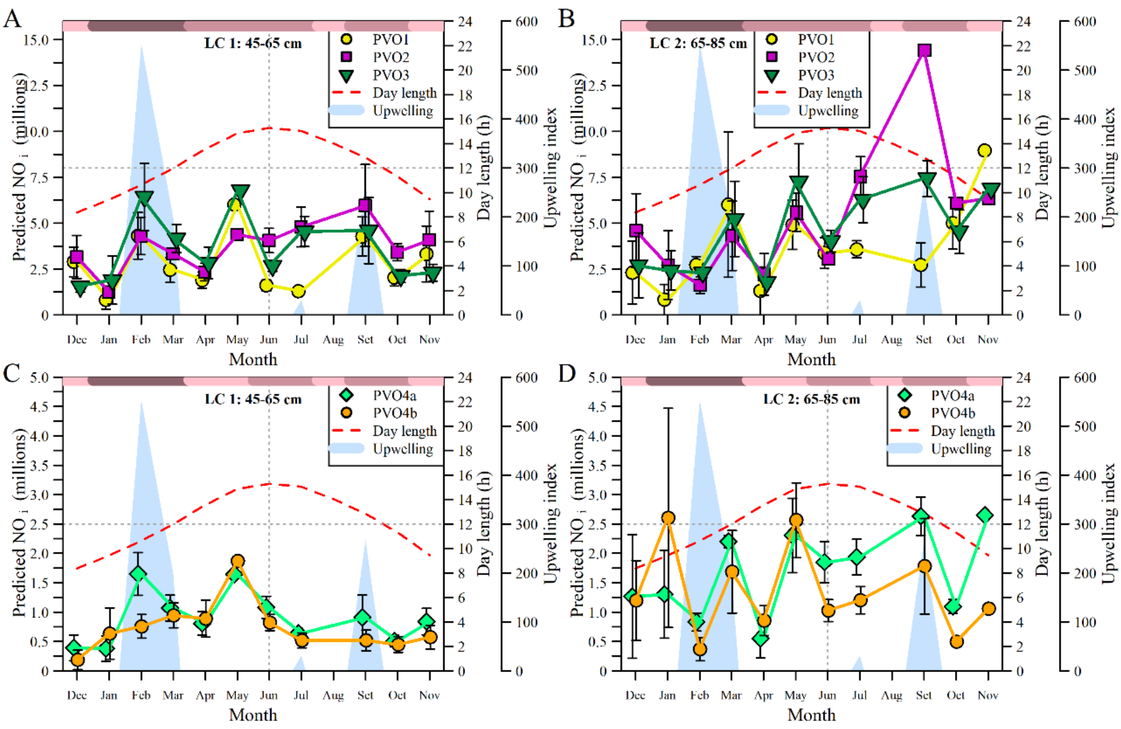

Fig. S7. Previtellogenic oocyte fluctuations, approach 3. Predicted stage$_i$ oocyte number (predicted NO$_i$) for (*A* and *B*) previtellogenic 1, 2 and 3 (PVO1, PVO2, PVO3) and (*C* and *D*) previtellogenic 4a and 4b (PVO4a and PVO4b) oocyte stages, split into two total length classes (LC): LC 1, from 45 to 65 cm (*A* and *C*) and LC 2 from 65 to 85 cm (*B* and *D*). The influences of total length and ovarian phase on fixed-gonad weight were modelled and thereby accounted for in the predictions. Estimates (mean ± SE) refer to data from European hake ovaries collected monthly at the Galician shelf from December 2011 to November 2012. Blue-shaded area shows upwelling events (upwelling index > 0) and dashed line day length. The upper box of the plot summarizes presently noticed spawning activity based on spawning fraction (SF) estimations: dark, medium and light purple corresponds to 30-50, 10-30 and <10% SF, respectively. The influence of total length and ovarian phase on gonad weight was accounted for.

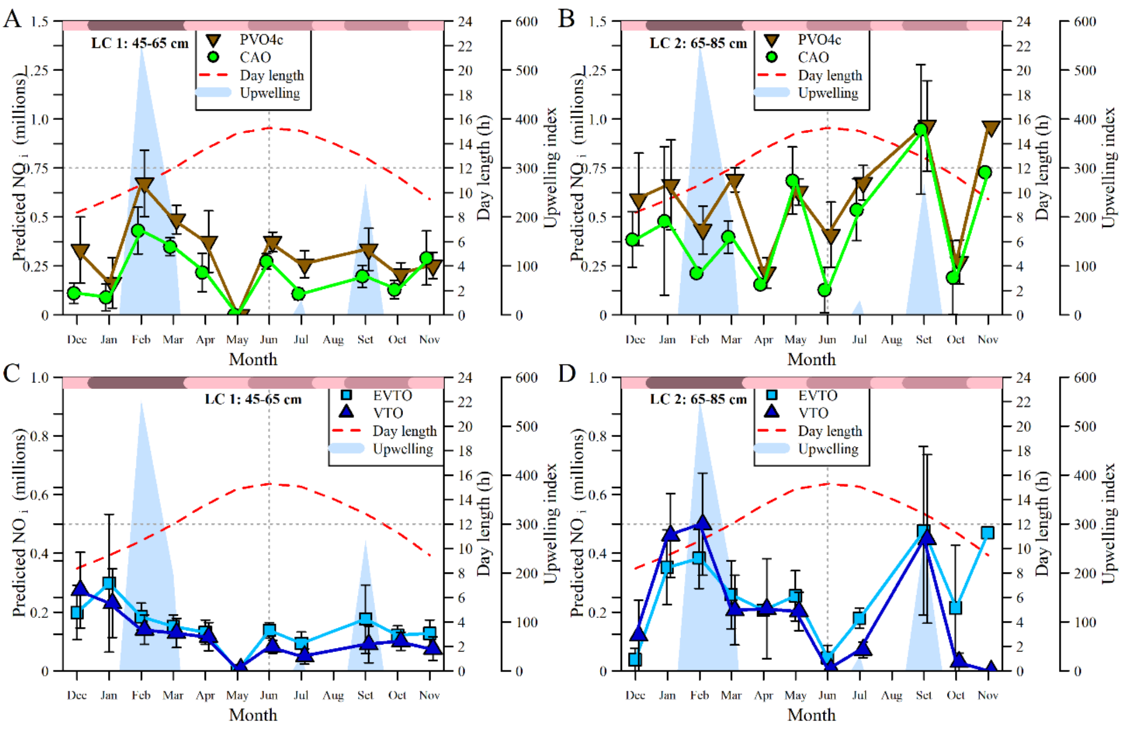

Fig. S8. Previtellogenic (PVO4c) and secondary growth oocyte fluctuations, approach 3. Predicted stage$_i$ oocyte number (predicted NO_i) for (*A* and *B*) previtellogenic 4c (PVO4c) and cortical alveoli (CAO) and (*C* and *D*) early vitellogenic (EVTO) and vitellogenic (VTO) oocyte stages, split into two total length classes (LC): LC 1, from 45 to 65 cm (*A* and *C*) and LC 2 from 65 to 85 cm (*B* and *D*). The influences of total length and ovarian phase on fixed-gonad weight were modelled and thereby accounted for in the predictions. Estimates (mean ± SE) refer to data from European hake ovaries collected monthly at the Galician shelf from December 2011 to November 2012. Blue-shaded area shows upwelling events (upwelling index > 0) and dashed line day length. The upper box of the plot summarizes presently noticed spawning activity based on spawning fraction (SF) estimations: dark, medium and light purple corresponds to 30-50, 10-30 and <10% SF, respectively. The influence of total length and ovarian phase on gonad weight was accounted for.

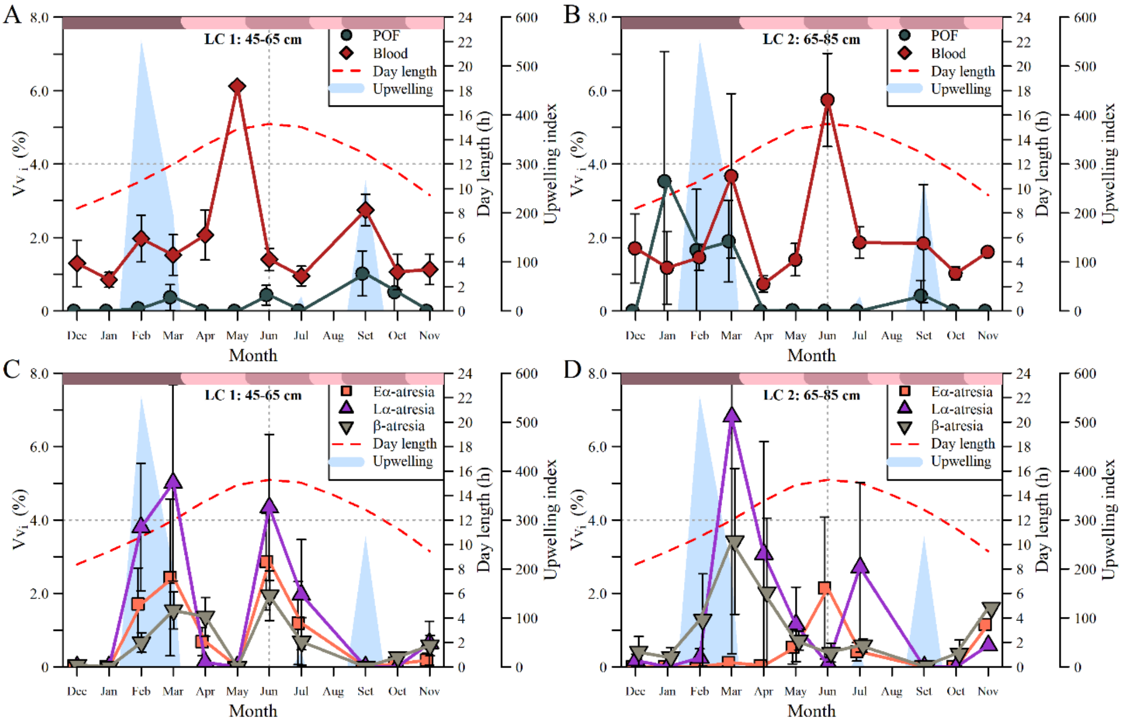

Fig. S9. Volume fraction of ovarian elements. Volume fraction (Vv_i) of (*A* and *B*) blood capillaries ("blood") and postovulatory follicles (POF) and (*C* and *D*) early-alpha (Eα), late-alpha (Lα) and β atresia, split into two total length classes (LC): LC 1, from 45 to 65 cm (*A* and *C*) and LC 2 from 65 to 85 cm (*B* and *D*). Estimates (mean ± SE) refer to data from European hake ovaries collected monthly at the Galician shelf from December 2011 to November 2012. Blue-shaded area shows upwelling events (upwelling index > 0) and dashed line day length. The upper box of the plot summarizes presently noticed spawning activity based on spawning fraction (SF) estimations: dark, medium and light purple corresponds to 30-50, 10-30 and <10% SF, respectively. The influence of total length and ovarian phase on gonad weight was accounted for.

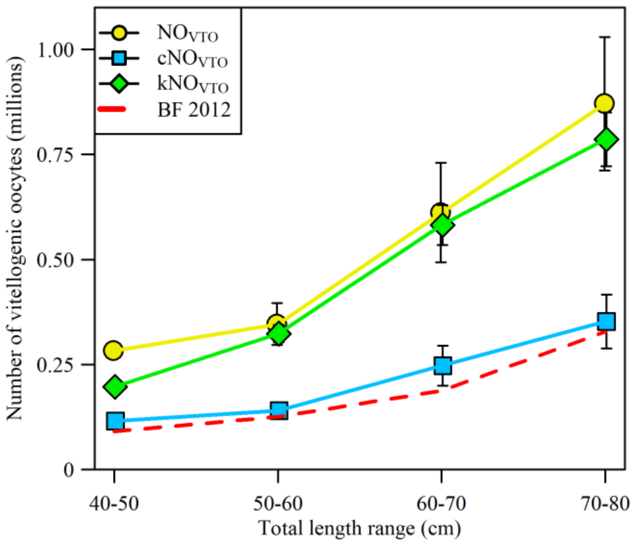

Fig. S10. Embedding-medium-related shrinkage correction of number of oocytes. Influences of embedding-medium-related shrinkage on OPD-based oocyte abundance estimates in southern European hake in 2012, using two types of correction factors: paraffin-embedded correction factor [74] and resin-embedded correction factor [21]. The resulting number of medium-late vitellogenic oocytes (VTO), non-corrected (NO_{VTO}) and corrected for shrinkage, either in paraffin (cNO_{VTO}) or resin (kNO_{VTO}), were compared with matching information on batch fecundity (BF) given from whole mount measurements. Mean ± SE are given.

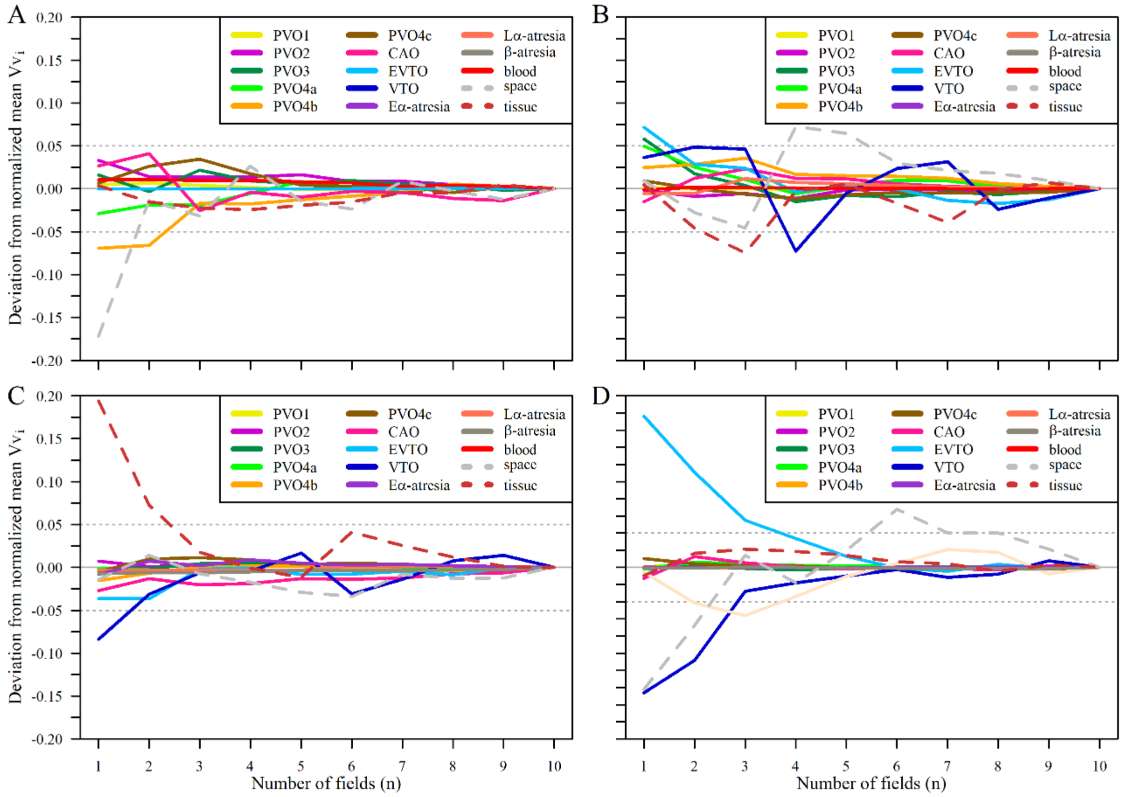

Fig. S11. Counting fields. Deviation from normalized grand mean of volume fraction (Vv_i), estimated by grid counting as a function of increasing number of counting fields (from 1 to 10) for four females in different ovarian phases (see *SI Materials and Methods*). Ovarian oocytes and additional elements considered: PVO1 (previtellogenic 1 oocytes), PVO2 (previtellogenic 2 oocytes), PVO3 (previtellogenic 3 oocytes), PVO4a (previtellogenic 4a oocytes), PVO4b (previtellogenic 4b oocytes), PVO4b (previtellogenic 4b oocytes), PVO4c (previtellogenic 4c oocytes), CAO (cortical alveoli oocytes), EVTO (early vitellogenic oocytes), VTO (medium and late vitellogenic oocytes), Eα-atresia (early-alpha atresia), Lα-atresia (late-alpha atresia), β-atresia (beta atresia), blood (blood capillaries), space (naturally empty space) and tissue (other organic elements).

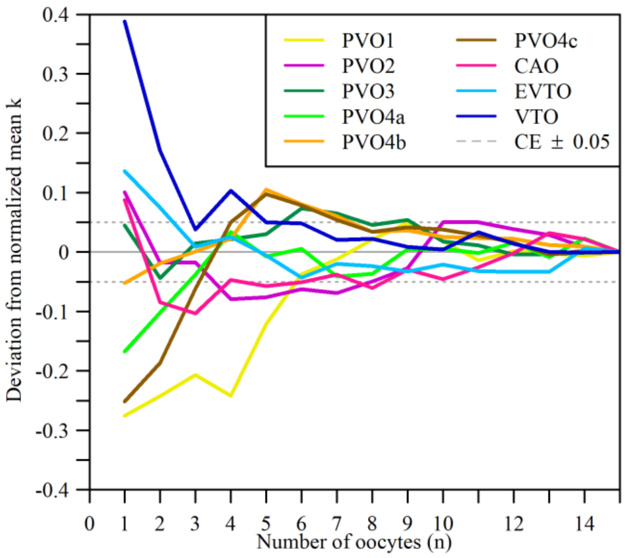

Fig. S12. Number of oocyte measurements. Deviation from normalized mean of stage$_i$ mean oocyte shape factor (k_i), estimated by considering increasing number of measurements (from 1 to 15) of single oocytes in different stages. Oocytes considered: PVO1 (previtellogenic 1 oocytes), PVO2 (previtellogenic 2 oocytes), PVO3 (previtellogenic 3 oocytes), PVO4a (previtellogenic 4a oocytes), PVO4b (previtellogenic 4b oocytes), PVO4b (previtellogenic 4b oocytes), PVO4c (previtellogenic 4c oocytes), CAO (cortical alveoli oocytes), EVTO (early vitellogenic oocytes), and VTO (medium and late vitellogenic oocytes). CE is coefficient of error.

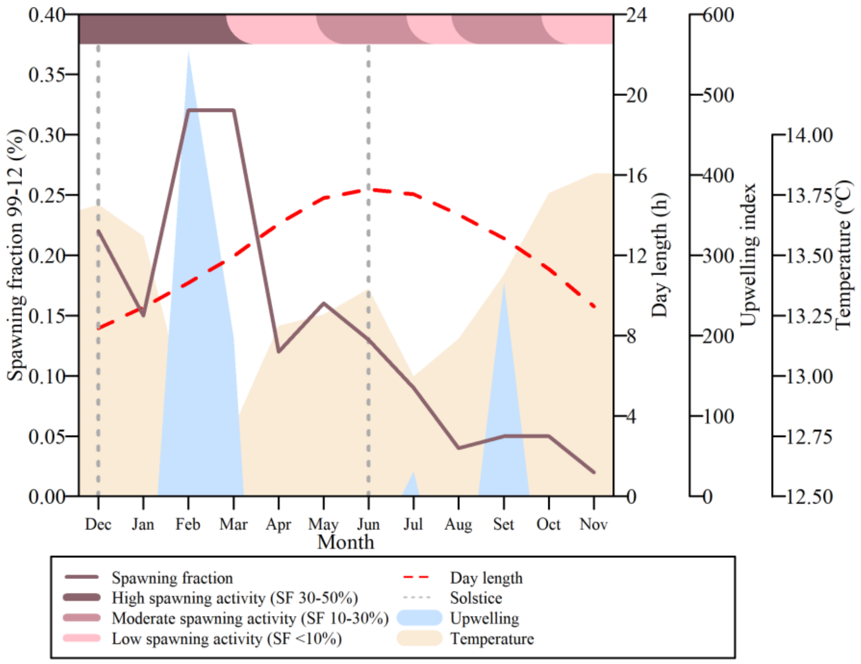

Fig. S13. **Environmental variables.** Monthly variation in mean values of the presently considered environmental variables along with spawning fraction of southern European hake (December 2011 – November 2012). Brown-shaded area shows sea water temperature from 50 to 350 m depth at 43.5ºN 9.5ºW, blue-shaded area upwelling events (upwelling index > 0), dashed line day length, and dotted line winter and summer solstice. The upper box of the plot summarizes presently noticed spawning activity based on spawning fraction (SF) estimations: dark, medium and light purple corresponds to 30-50, 10-30 and <10% SF, respectively. Solid line depicts overall SF in the same waters from 1999 to 2012.

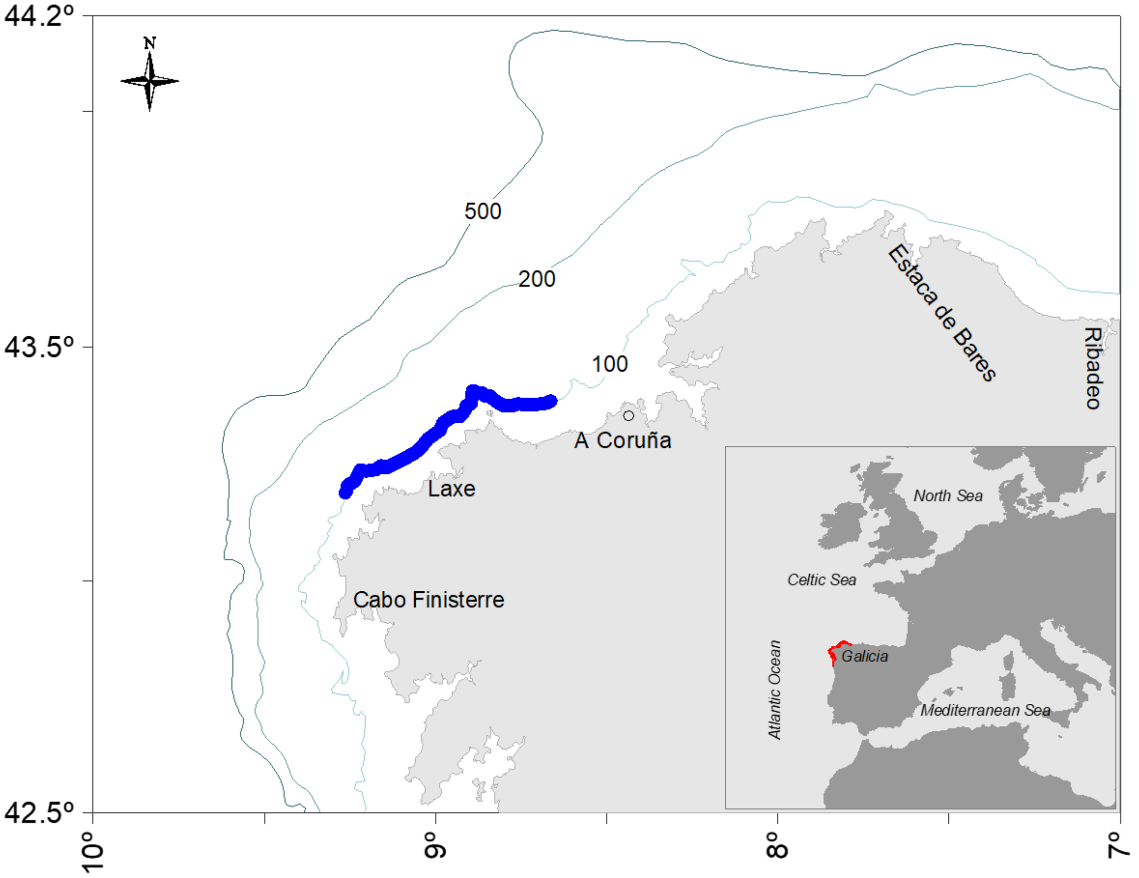

Fig. S14. Study area. Map showing the Galician shelf (inserted box, in red), where the presently examined females of European hake were sampled in the period from December 2011 to November 2012 (blue area). Bathymetry is indicated as three contour lines (in meter).

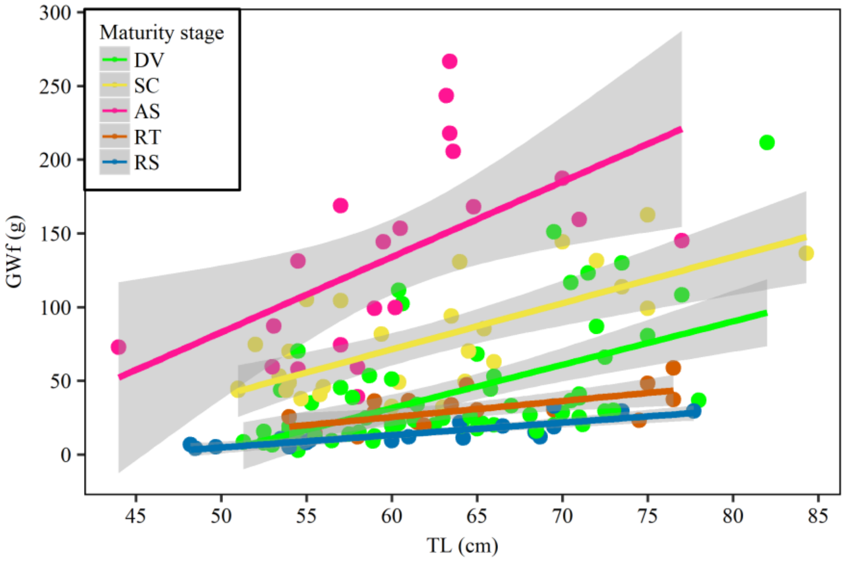

Fig. S15. Relationship between gonad weight and fish size. Formalin-fixed gonad weight (GW_f) as a function of total length (TL) at different maturity stages (ovarian phases; MAT) in southern European hake, given from Generalized linear model runs. Shaded region refers to the 95% confidence band. DV: developing; SC: spawning capable; AS: actively spawning; RT: regenerating; RS: regressing females.

Table S1. **Sampling scheme.** Sampling scheme in the period from December 2011 to November 2012, giving the number of southern European hake females analysed by OPD theory by quarter and ovarian phase (MAT). For each phase the corresponding most advanced oocyte stage (MAO), presence and age (recent vs. old) of postovulatory follicles (POFs) and atresia (either α- or β-atresia) are shown. PVO: previtellogenic; CAO: cortical alveoli; EVTO: early vitellogenic; VTO: medium and late vitellogenic; MNO: migratory nucleus; HYO: hydrated oocytes.

**Maturity**		**Quarter**[a]				
**MAT (ovarian phase)**	**MAO (oocyte stage)**	**1**	**2**	**3**	**4**	**Total**
IM-Immature	PVOs		3		6	9
DV-Developing	CAO /EVTO/VTO	14	29	14	9	66
SC-Spawning capable	MNO (POFs)	13	5	7	4	29
AS-Actively spawning	HYO (recent POFs)	12	5	3	2	22
RS-Regressing	α-atresia (old POFs)	7	7	3	1	18
RT-Regenerating	PVOs, β-atresia		5	8	5	18
	**TOTAL**	**46**	**54**	**35**	**27**	**162**

[a] **Note**: December 2011 is included in Quarter 1, while Quarter 4 accounts only for October and November 2012.

Table S2. Histological descriptions. Terminology of histological features. Histological description of various ovary elements seen under the light microscope, split into oocytes in different development stages and other, relevant ovary structures detected.

**Ovary elements**	**Histological features**	**OD (µm)[a]**	**OD (µm)[b]**
OOCYTES			
Previtellogenic 1 (PVO1)	Cytoplasm stains weakly and homogenously	19 ± 2	53±4
Previtellogenic 2 (PVO2)	Cytoplasm stains stronger and irregularly; small white areas; RNA spreading from the perinuclear zone	42 ± 5	81±6
Previtellogenic 3 (PVO3)	Cytoplasm stains stronger and irregularly; small white areas; organelles/RNA distributed homogeneously throughout the entire cytoplasm	68 ± 7	121±10
Previtellogenic 4a (PVO4a)	Slightly distinctive circumnuclear ring (CNR) (darker reticulate structure) occupying almost the whole cytoplasm; only a small area adjoining the membrane remains homogeneous and lighter	90 ± 11	151±15
Previtellogenic 4b (PVO4b)	CNR very distinct and centrally located in the cytoplasm, separated from the nucleus and the membrane by a layer of homogeneous light cytoplasm	108 ± 15	175±19
Previtellogenic 4c (PVO4c)	CNR gradually disappears towards the periphery; follicular layer becomes evident, some vacuolization, follicular envelopment evident	130 ± 14	207±29
Cortical alveoli (CA)	Vacuolization of the cytoplasm, monolayer chorion that stains stronger	169 ± 16	259±23
Early vitellogenic (EVTO)	Small yolk granules begin to accumulate in a narrow peripheral area of the cytoplasm; double-layered chorion (inner layer thinner than outer layer)	246 ± 30	370±46
Medium/late vitellogenic (VTO)	Larger yolk granules accumulate throughout the whole cytoplasm; inner layer of the chorion becomes equal or wider than the outer layer	433 ± 64	623±112
Migratory nucleus (MN)	Oil droplets forming a single vesicle "pushing" the germinal vesicle towards the animal pole; yolk granules start to fuse	556 ± 103	793±112
Hydrated (HY)	Germinal vesicle no longer visible; homogeneous cytoplasm, increase in oocyte size due to intake of water (but collapsing during histological processing)	NA	NA
OTHER STRUCTURES			

Early-α atretic oocyte (Eα)	Thin breaks appear in the chorion, cytoplasm disorganized
Late-α atretic oocyte (Lα)	Wider breaks in the chorion, fragments moving inwards
β atretic oocyte (β)	No chorion left, no clear yolk content visible, high degree of vacuolization
Missing oocyte	Empty oocyte space
Postovulatory follicle (POF)	Follicular envelop after ovulation
Other tissue	Ovary wall, connective tissue, etc.
Blood	Blood capillaries
Empty	Natural empty space (lumen) inside the ovary
Out	Outside the ovary wall

Note: Mean oocyte diameter (OD ± SD) is given, where [a] is non-corrected and [b] corrected for shrinkage, applying correction factors given for paraffin-embedded material (10).

Table S3. ANCOVA table for the variance in formalin-fixed gonad weight (GW_f) with total length (TL) at different ovarian phases (MAT).

	**Type III SS**	**Mean SS**	**df**	**F**	**P**
**TL**	41202	41202	1	37.44	<0.001
**MAT**	226931	56733	4	51.55	<0.002
**TL × MAT**	13559	3390	4	3.08	<0.003
**Residuals**	156276	1101	142		
			**Adjusted R^2 = 0.6202**		

S1 Text. Supplementary results. Selection of the method to estimate total oocyte numbers.

Supplementary Results

Selection of the method to estimate total oocyte numbers. When converting OPD_i values to corresponding absolute $stage_i$ oocyte numbers (NO_i) in the whole ovary, one should be aware of that the latter is dependent on both ovarian phase and body size, as already handled by Schismenou et al. [22] statistically (GLM) and therefore done likewise here. We evaluated thereafter the appropriateness of the above-mentioned three different methods to estimate oocyte productivity, aiming at using only one of them to study the seasonal dynamics, focusing especially on reducing the variability associated with body size dependency while undertaking as little data transformations as possible. To test the methods and for the sake of simplicity only PVO4a-b oocytes were firstly considered. As shown in the main text, these are key stages in relation to early oocyte recruitment.

- *Approach 1 (Untransformed and Length-Grouped Data Sets).* As expected, the number of oocytes were higher in larger than smaller females (S1A,C Fig), but also the seasonal variation of NO_i differed between length classes (LC). In more details, in LC 1 (TL: 45-65 cm) NO_i decreased from the first to the second half of the year, while in LC 2 (TL: 65-85 cm) PVO4a showing indications of an increase and PVO4b a decrease. Corresponding analyses for other oocyte stages were undertaken as well (S3 and S4 Fig) finding, in general, that NO_i based on this approach showed increased variability in LC 2.
- *Approach 2 (Relative TL-Based NO_i).* In contrast to the above settings, this method provided patterns independent of female lengths, except for PVO1, 2 and 3 which were more abundant in LC 1 (S5 and S6 Fig). The seasonal variation in NO_i (S1C Fig) using this approach showed relatively larger numbers from March to May decreasing

afterwards for PVO4b, but flattening out for PVO4a. Hence, this described overall pattern as such could be said to mirror that one seen for LC 1 in Approach 1 (S1A *vs*. S1C Fig), the length class with nearly twice as many females analysed (see above).

- *Approach 3 (Predicted NO_i using Model-Based Gonad Weight)*. This approach produced generally a lower variability and standard error (S1D Fig) compared to Approaches 1 and 2, but still showed differences between length classes (S7 and S8 Fig), rather similarly to Approach 1.

As Approach 2 was the only method providing results independent of female size, this method was selected in the subsequent analyses to assess the *overall*, temporal trends in mean number of $stage_i$ oocytes. This analysis was also encouraged by the presence of isometric body growth, i.e. the exponent b could be safely set at 3, i.e. TL^3, as used in the denominator in the EW vs. TL formula in question (cf. S14A Fig.). Further to this, due to the higher resilience of TL, the use of this metric instead of EW spoke for a reduced, sporadic variability in NO_i.

S2 Text. Supplementary discussion. Technical and methodological considerations about the quantification of early oocytes.

Supplementary Discussion

Technical and Methodological Considerations about the Quantification of Early Oocytes. Logically, quantifying these small cells involves the risk of significant underestimations by simple techniques, such as profile (2-D) counting in histological slides [77]. One of the first steps in any stereological (3-D) work is to decide upon which grid to apply. Here the work of Seivåg et al. [78] was found particularly relevant, addressing very small objects represented by sperm cells in the bearded goby (*Sufflogobius bibarbatus*), using a fine resolution version of the standard Weibel grid [79], i.e. 418 instead of 256 maximum number of hits within each defined observational frame (field). We landed on a grid version close to the former (370 hits per field) to assure precise estimations but at reasonable labor efforts. Biases due to heterogeneity of the distribution of oocytes within the ovary, especially notable at some ovarian phases, was minimized by counting seven fields per female, covering the whole ovarian cross section. As the adopted OPD equation is sensitive to small changes in oocyte size and shape [22,74], seen naturally with the gradual development of the ovary, ten stage$_i$ oocytes were carefully classified and measured in each female. Thus, the presence of hydrated oocytes, all collapsed, was only noted. This former practice implied that each oocyte type showed little intrinsic variation in morphological descriptors (as oocyte diameter or volume) but, potentially, in numbers across females. Other technical aspects such as shrinkage during histological processing likely play a stronger impact towards final oocyte maturation, i.e. during accelerated water uptake [74]. More specifically, Korta et al. [21] calculated the correction factor for samples embedded in resin, while we used paraffin as such medium, which generally introduces more shrinkage [74]. We opted for using the relevant correction

factor in the latter dedicated study, being established for the tuna albacore showing a comparable, complex reproductive style. Further to this, it was confirmed that OPD-based 95% confidence intervals safely overlap with those based on manual counting, tested on migratory-nucleus oocytes [80]. The great prospects of the OPD method in the present context with multiple cell types present, although being theoretically based, were demonstrated in a previous study [22] finding close matches between resin-OPD- and whole-mount-digital-based VTO estimates.
